# Neutralizing Antibody Induction by HIV-1 Envelope Glycoprotein SOSIP Trimers on Iron Oxide Nanoparticles May Be Impaired by Mannose Binding Lectin

**DOI:** 10.1128/JVI.01883-19

**Published:** 2020-02-28

**Authors:** Rajesh P. Ringe, Victor M. Cruz Portillo, Pia Dosenovic, Thomas J. Ketas, Gabriel Ozorowski, Bartek Nogal, Lautaro Perez, Celia C. LaBranche, Jillian Lim, Erik Francomano, Ian A. Wilson, Rogier W. Sanders, Andrew B. Ward, David C. Montefiori, Michel C. Nussenzweig, P. J. Klasse, Albert Cupo, John P. Moore

**Affiliations:** a Department of Microbiology and Immunology, Weill Cornell Medical College, New York, New York, USA; b Laboratory of Molecular Immunology, The Rockefeller University, New York, New York, USA; c Department of Integrative Structural and Computational Biology, Consortium for HIV Vaccine Development (CHAVD), The Scripps Research Institute, La Jolla, California, USA; d Duke University Medical Center, Durham, North Carolina, USA; e Department of Medical Microbiology, Amsterdam University Medical Centers, Amsterdam, The Netherlands; f Howard Hughes Medical Institute, The Rockefeller University, New York, New York, USA; Emory University

**Keywords:** HIV-1 immunogens, nanoparticles, SOSIP trimer, mannose binding lectin, neutralizing antibodies

## Abstract

We covalently attached human immunodeficiency virus type 1 (HIV-1) Env SOSIP trimers to iron oxide nanoparticles (IO-NPs) to create a particulate immunogen for neutralizing antibody (NAb) induction. The attached trimers, ∼20 per particle, retained native-like antigenicity, judged by reactivity with NAbs and non-NAbs. Bivalent (BG505 and B41) trimer IO-NPs were made, as were IO-NPs displaying B41 trimers carrying a PADRE T-cell helper epitope (TCHE). We immunized mice with B41 soluble or IO-NP trimers after PADRE peptide priming. After two immunizations, IO-NP presentation and the TCHE tag independently and substantially increased anti-trimer antibody responses, but titer differences waned after two further doses. Notable and unexpected findings were that autologous NAbs to the N289 glycan hole epitope were consistently induced in mice given soluble but not IO-NP trimers. Various recombinant mannose binding lectins (MBLs) and MBLs in sera of both murine and human origin bound to soluble and IO-NP trimers. MBL binding occluded the autologous NAb epitope on the B41 IO-NP trimers, which may contribute to its poor immunogenicity. The exposure of a subset of broadly active NAb epitopes was also impaired by MBL binding, which could have substantial implications for the utility of trimer-bearing nanoparticles in general and perhaps also for soluble Env proteins.

**IMPORTANCE** Recombinant trimeric SOSIP proteins are vaccine components intended to induce neutralizing antibodies (NAbs) that prevent cells from infection by human immunodeficiency virus type 1 (HIV-1). A way to increase the strength of antibody responses to these proteins is to present them on the surface of nanoparticles (NPs). We chemically attached about 20 SOSIP trimers to NPs made of iron oxide (IO). The resulting IO-NP trimers had appropriate properties when we studied them in the laboratory but, unexpectedly, were less able to induce NAbs than nonattached trimers when used to immunize mice. We found that mannose binding lectins, proteins naturally present in the serum of mice and other animals, bound strongly to the soluble and IO-NP trimers, blocking access to antibody epitopes in a way that may impede the development of NAb responses. These findings should influence how trimer-bearing NPs of various designs are made and used.

## Abstract (precis)

Recombinant trimeric SOSIP proteins are vaccine components intended to induce neutralizing antibodies (NAbs) that prevent cells from infection by human immunodeficiency virus type 1 (HIV-1). A way to increase the strength of antibody responses to these proteins is to present them on the surface of nanoparticles (NPs). We chemically attached about 20 SOSIP trimers to NPs made of iron oxide (IO). The resulting IO-NP trimers had appropriate properties when we studied them in the laboratory but, unexpectedly, were less able to induce NAbs than nonattached trimers when used to immunize mice. We found that mannose binding lectins, proteins naturally present in the serum of mice and other animals, bound strongly to the soluble and IO-NP trimers, blocking access to antibody epitopes in a way that may impede the development of NAb responses. These findings should influence how trimer-bearing NPs of various designs are made and used.

## INTRODUCTION

A vaccine to prevent human immunodeficiency virus type 1 (HIV-1) infection will most likely need to induce potent and broad neutralizing antibodies (NAbs) that recognize the gp120 plus gp41 envelope glycoprotein trimer on the virion surface (1–5). The fragile virion-associated trimer can be appropriately mimicked for vaccine development by soluble recombinant SOSIP trimers that are stabilized by engineered sequence changes and that are produced in amounts sufficient for animal and human studies (3, 4, 6, 7).

Compared to many other pathogen antigens, HIV-1 Env proteins in general are poorly immunogenic even with adjuvants (8, 9). Env immunogens induce only weak and transient serum antibody (Ab) responses, and broadly active NAbs (bNAbs) against multiple strains emerge during HIV-1 infection only after extensive somatic hypermutation of precursors (10–12). Here, we explore strategies to improve NAb responses by presenting trimers on iron oxide nanoparticles (IO-NPs) and/or including an exogenous T-cell helper epitope (TCHE).

Multivalent antigen display on 25- to 50-nm NPs may drive stronger, longer-lasting Ab responses via efficient cross-linking of B-cell receptors (BCR) and improved antigen trafficking and presentation (13–16). The human papillomavirus vaccine involves NPs, as do cutting-edge programs to create influenza virus and respiratory syncytial virus (RSV) vaccines (13, 17–20). Only limited benefits to NAb titers were seen when early-generation HIV-1 Env proteins were tested as NPs, which may reflect design limitations (e.g., NP instability *in vivo* and poor Env epitope display) (15, 21–25). Recent reports on SOSIP trimer-based NPs provide insights into how epitope location affects immunogenicity and how innate immune factors, notably, mannose binding lectins (MBLs), influence NP trafficking to follicular dendritic cells and germinal centers (15, 16, 26, 27).

One SOSIP trimer-NP design involves self-assembling, protein-only ferritin or I53-50 nanocages (18, 26–29). Alternatively, trimers can be linked noncovalently or covalently to the surface of preexisting NPs, such as, but not limited to, liposomes (15, 22, 24, 30). Here, we describe the production, *in vitro* properties, and immunogenicity of IO-NPs of 24 nm in diameter to which ∼20 SOSIP trimers were covalently linked via surface lysine residues. An IO-NP-based malaria parasite protein vaccine was safe in a human clinical trial (31), and IO-NPs are used to deliver cancer therapeutics (32–34).

We also assessed whether exogenous TCHEs could improve SOSIP trimer immunogenicity. The availability of the few TCHEs in the HIV-1 Env sequence for major histocompatibility complex class II presentation may be highly restricted by glycans and disulfide bonds; glycans can be present within a TCHE sequence or interfere with Env protein processing and, hence, the liberation of TCHE-containing peptides (35–42). As the topic is underresearched for modern Env protein designs, we incorporated a TCHE into SOSIP trimers as a C-terminal PADRE tag flanked by cathepsin S cleavage sites intended to facilitate its release.

For an initial evaluation of immunogenicity, we immunized mice with soluble and IO-NP SOSIP trimers of the B41 (clade B) genotype that either included or lacked the PADRE tag. Both IO-NP presentation and the TCHE tag initially increased anti-trimer Ab titers, but the differentials waned subsequently. Autologous NAbs to a glycan hole epitope were induced by soluble trimers, which was unexpected, as NAbs to a tier 2 virus have rarely been raised previously in mice (21, 43–45). However, the autologous NAb responses to the IO-NP trimers were much weaker, which was also unanticipated. In follow-up studies *in vitro*, the binding of murine recombinant and serum MBLs to IO-NP trimers occluded the autologous NAb epitope and also a subset of bNAb epitopes located in the lower half of the trimer. Human MBL was also trimer reactive. We suggest that serum MBLs may limit the immunogenicity of certain epitopes on SOSIP trimers, which could have substantial implications for how immunogens of various designs are used.

## RESULTS

### Presenting SOSIP trimers on chemically functionalized IO-NPs.

**FIG 1:**
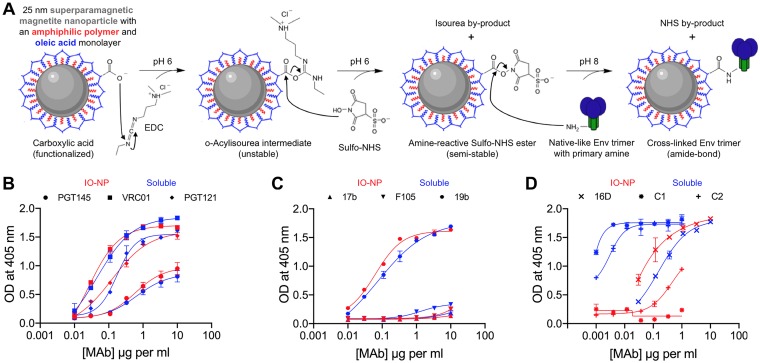
Production and antigenicity of B41 SOSIP.v4.1 IO-NP trimers. (A) Surface carboxylate groups on lipid-coated IO-NPs were treated with EDC and sulfo-NHS cross-linking agents. SOSIP trimers were coupled to the activated particles via surface amine groups at pH 8 to create IO-NP trimers. (B) 2G12-capture ELISA analyses of soluble (blue lines and symbols) and IO-NP (red lines and symbols) B41 SOSIP.v4.1 trimers with bNAbs PGT145, VRC01, and PGT121. (C) As in panel B but with non-NAbs 17b, F105, and 19b. (D) As in panel B but with rabbit NAb 16D and non-NAbs C2 and C1.

Key requirements for a particulate antigen include stability *in vitro* and *in vivo* and a practical production process (13, 14, 25, 27). SOSIP trimers must retain their native-like properties when attached to NPs. Covalently linking SOSIP trimers to liposome NPs is superior to noncovalent attachment (21, 25). Highly regular, spherical, 24-nm-diameter IO-NPs comprise an iron oxide core stably coated with an oleic acid derivative to which proteins or peptides can be covalently cross-linked under mild conditions (neutral pH, low salt, room temperature) (31, 33). Briefly, the amine moieties of the lysines on the SOSIP trimer surface react with carboxylate moieties on the oleic acid coating before magnetic separation of IO-NP-bound and uncoupled trimers (Fig. 1A).

### Biochemical properties of SOSIP trimer IO-NPs.

Twenty closely packed SOSIP trimers are presented on the surface of a self-assembling I53-50 NP core of ∼25 nm in diameter (26, 27). We sought to couple a similar average number of trimers to each 24-nm-diameter IO-NP. Protein quantitation and the manufacturer-provided assessment of the number of IO-NPs per milligram guided initial coupling efficiency assessments when input concentrations of BG505 and B41 SOSIP trimers were varied. We found that we could attach 15 to 20 BG505 SOSIP.664 or B41 SOSIP.v4.1 trimers per IO-NP, but the highest stoichiometry consistently achievable for BG505 SOSIP.v4.1 was ∼9 (Table 1).

**TABLE 1:** Stoichiometry of SOSIP trimer attachment to IO-NPs

Trimer designation	No. of trimers coupled per IO-NP^*a*^
B41 SOSIP.v4.1	13–15
B41 SOSIP.v4.1-N289-KI	15–18
B41 SOSIP.v4.1-PADRE-v2	12–15
B41 SOSIP.v4.1-PADRE-v3	15–20
B41 SOSIP-E64K.M1M7	18–20
B41 SOSIP.v4.1-KG4	18–20
BG505 SOSIP.664	18–20
BG505 SOSIP.v4.1	7–9
BG505 SOSIP.v4.1-KG4	20–25
B41 SOSIP.v4.1-KG4 + BG505 SOSIP.v4.1-KG4	18–20
BG505 SOSIP.v4.1-GT1.1	12–15
BG505 SOSIP-E64K.M1M7	18–20
CZA97 SOSIP.v4.2-M6.IT-KG4	20–22
16055 SOSIP.v8-KG4	18–20

a The average number of various SOSIP trimers attached to each IO-NP was estimated as described in Materials and Methods.

**FIG 2:**
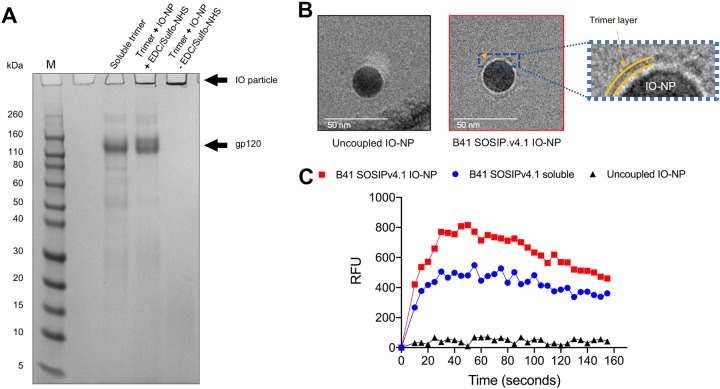
Reducing SDS-PAGE gel analysis of B41 soluble and IO-NP trimers, cryo-EM images of IO-NPs, and B-cell activation by IO-NPs. (A) SDS-PAGE gel of B41 SOSIP.v4.1 soluble and IO-NP trimers, as indicated. When sulfo-NHS and EDC were omitted, the soluble trimer was not covalently coupled to the IO-NPs (right lane). Exposure to SDS and DTT presumably dissolves the oleic acid coating on the particles and liberates the attached trimers. The positions where IO particles and gp120 migrate are shown, as are marker proteins (lane M), with the molecular masses indicated in kilodaltons. The IO-NPs do not enter the gel, but Coomassie blue-stained, released gp120 subunits migrate at the same position as those from similarly treated soluble trimers. (B) Zero-defocus cryo-EM images of uncoupled (left) and B41 SOSIP.v4.1 trimer-coupled (middle) IO-NPs. The cores of the 24-nm-diameter, monodisperse IO-NPs are clearly visible, but visualization of the surface proteins was challenging, as iron atoms not only scatter electrons but also diffract the electron beam. On the right is a zoom in of the region indicated by the yellow arrow in the middle image, to emphasize a fuzzy gray ring (yellow bands) surrounding the IO-NP core that is visible only on the trimer-bearing IO-NPs. We were unable to assess the orientation or stoichiometry of the attached trimers. (C) Ramos B cells expressing the VRC01 bNAb BCR were stimulated with 10 μg of the B41 SOSIP.v4.1 soluble (blue) or IO-NP (red) trimer or the equivalent amount of uncoupled IO-NPs (black). The recorded fluorescence outputs are representative of those from two experiments. RFU, relative fluorescence units.

IO-NP trimers were routinely analyzed by reducing SDS-polyacrylamide gel electrophoresis (PAGE). In the absence of the coupling agents, the trimers did not attach to the particles (Fig. 2A). Negative-stain electron microscopy (NS-EM) was not feasible. as iron atoms scatter electrons similarly to heavy atom stains, impairing the contrast between particles and background, but we obtained low-resolution cryo-electron microscopy (cryo-EM) images of B41 SOSIP.v4.1 trimer IO-NPs (Fig. 2B). The same IO-NP trimers triggered Ca^2+^ signaling more strongly than their soluble counterparts in B cells that express the VRC01 bNAb (Fig. 2C). Other trimer-NP designs do the same (21, 25, 26).

### Antigenicity of B41 SOSIP.v4.1 soluble and IO-NP trimers by ELISA.

To compare the antigenicity of B41 SOSIP.v4.1 soluble and IO-NP trimers, we used an enzyme-linked immunosorbent assay (ELISA) in which bNAb 2G12-captured antigens were probed with biotin-labeled monoclonal antibodies (MAbs) against various epitope clusters. The bNAbs PGT145 (V2 apex), VRC01 (CD4 binding site [CD4bs]), and PGT121 (V3 glycan) bound comparably to IO-NP and soluble trimers (Fig. 1B), whereas non-NAbs F105 (CD4bs) and 17b (CD4 induced [CD4i]) reacted poorly in both cases (Fig. 1C). The 19b non-NAb (V3) bound strongly to both soluble and IO-NP trimers (Fig. 1C), reflecting the artifactual exposure of V3 epitopes on SOSIP trimers under capture ELISA conditions (see below) (46–49). Three MAbs isolated from B41 SOSIP trimer-immunized rabbits were tested: 16D, an autologous NAb specific to the N289 glycan hole epitope bound efficiently to both soluble and IO-NP trimers. However, non-NAbs C1 and C2 against neoepitopes on the soluble trimer base reacted poorly or not detectably with the IO-NP trimers (Fig. 1D).

Overall, B41 SOSIP.v4.1 IO-NP trimers retained the favorable bNAb-versus-non-NAb antigenicity profile of soluble trimers. The differential binding of C1 and C2 shows that the normally accessible and potentially immunodistractive trimer base area is substantially occluded after coupling to IO-NPs.

**FIG A1:**
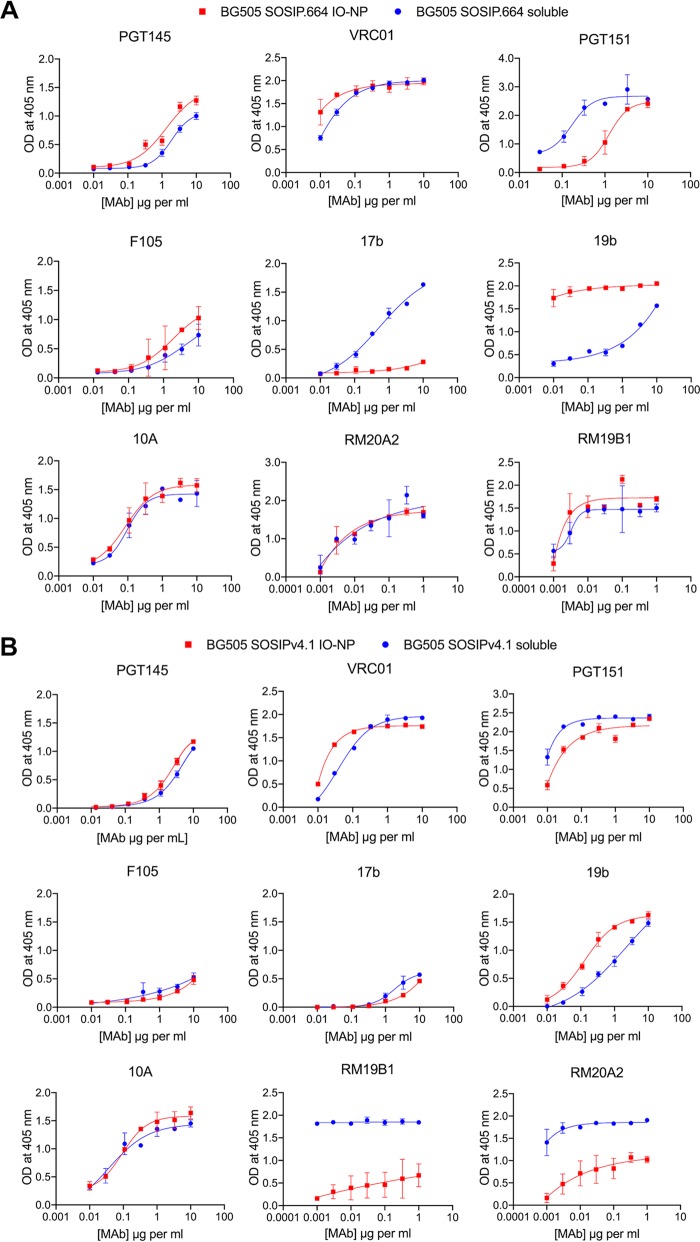
Antigenicity of BG505 SOSIP.664 and SOSIP.v4.1 soluble and IO-NP trimers by a 2G12-capture ELISA. (A) Soluble (blue symbols) and IO-NP (red symbols) BG505 SOSIP.664 trimers were tested for binding by bNAbs PGT145, VRC01, and PGT151; non-NAbs 17b, F105, and 19b; rabbit anti-glycan hole NAb 10A; and macaque anti-base non-NAbs RM20A2 and RM19B1. (B) As for panel A, but using BG505 SOSIP.v4.1 trimers.

**FIG A2:**
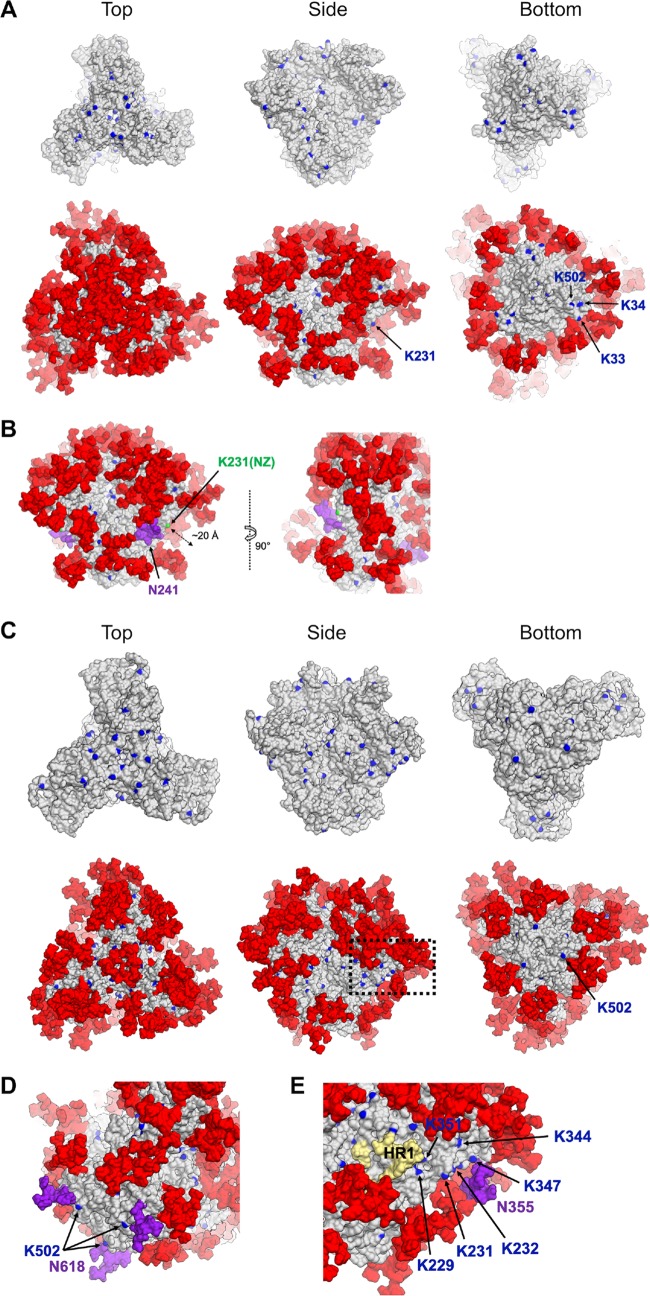
Surface lysine residues on B41 and BG505 SOSIP.v4.1 trimers. (A) B41 SOSIP.v4.1 trimers are viewed from the top, side, and bottom, as indicated. The upper row shows the peptidic surface with lysines colored blue. In the lower row, Man_9_ glycans (red) are added (lighter red shading indicates more distant glycans). For simplicity, all PNGS are considered to be occupied by a Man_9_ oligomannose moiety (see Materials and Methods). The lysine residues mentioned in the text are labeled. (B) More detailed representation of the environment near lysine-231 (green), with the N241 glycan highlighted in purple. (C) The model shows BG505 SOSIP.664 trimers viewed from the top, side, and bottom, as indicated. The color scheme and layout are as described in the legend to panel A. The lysine-502 residue is labeled. (D) A more detailed representation of the environment near BG505 lysine-502 (blue), with the N618 glycan being highlighted in purple. (E) Detail of a lysine-rich patch in the BG505 gp120 C2 and C3 regions, with gp41 HR1 in yellow and N355 glycan in purple. This area of the trimer is boxed in the side view of the trimer in panel C.

A similar antigenicity analysis was conducted on IO-NP trimers displaying BG505 SOSIP.664 or SOSIP.v4.1 trimers (see Fig. A1). Taken together with stoichiometry and modeling data, the antigenicity profiles imply that BG505 SOSIP.664 trimers couple to IO-NPs efficiently but in an orientation(s) whereby the base is at least partially exposed and the apex is less than fully accessible; in simple terms, a proportion of these trimers may be attached upside down. In contrast, their SOSIP.v4.1 counterparts attached less well but did so predominantly via their base, most likely because the lysines near the trimer apex are less accessible as a result of the additional stabilizing changes present in the SOSIP.v4.1 design (Table 1; see Fig. A1 and A2).

### Preferential attachment of SOSIP trimers to IO-NPs via an engineered, lysine-rich tag.

**FIG 3:**
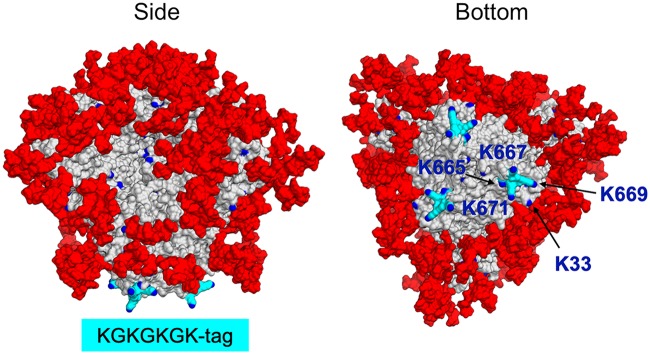
A lysine-rich tag added to the base of B41 SOSIP.v4.1 trimers. The location of the KG4 (665-KGKGKGK-671) C-terminal tags (colored cyan) on the B41 SOSIP.v4.1 trimer base, viewed from the side and bottom, is shown. Lysines are in blue and labeled (K33 is endogenous to the B41 sequence). Man_9_ glycans are colored red.

**FIG A3:**
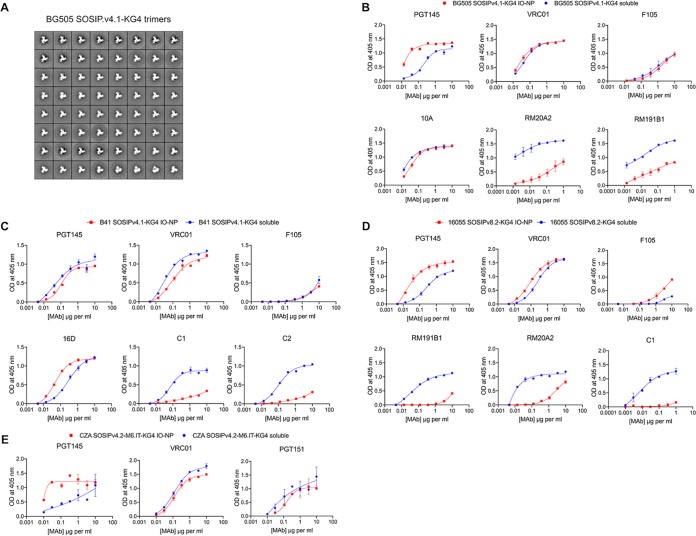
Antigenicity of KG4-tagged soluble and IO-NP trimers by ELISA. (A) NS-EM images show that the soluble BG505 SOSIP.v4.1-KG4 trimer is 100% native-like. (B) Results of a 2G12-capture ELISA with soluble (blue symbols) and IO-NP (red symbols) BG505 SOSIP.v4.1-KG4 trimers using bNAbs PGT145 and VRC01; non-NAb F105; rabbit anti-glycan hole NAb 10A; and macaque anti-base non-NAbs RM20A2 and RM19B1. (C) Results of a 2G12-capture ELISA with soluble (blue symbols) and IO-NP (red symbols) B41 SOSIP.v4.1-KG4 trimers using bNAbs PGT145 and VRC01, non-NAb F105, and rabbit MAbs 16D, C1, and C2, as indicated. (D) Results of a 2G12-capture ELISA with soluble (blue symbols) and IO-NP (red symbols) 16055 SOSIP.v8-KG4 trimers using bNAbs PGT145 and VRC01, non-NAb F105, and rabbits MAbs RM20A2, RM19B1, and C1. (E) Results of a 2G12-capture ELISA with soluble (blue symbols) and IO-NP (red symbols) CZA97 SOSIP.v4.2-M6.IT-KG4 trimers using bNAbs PGT145, VRC01, and PGT151.

As noted above, SOSIP trimers are coupled to IO-NPs via the amine moieties of surface-accessible lysine residues (Fig. 1A). Models of the B41 and BG505 SOSIP trimers show that most surface lysines are shielded by glycans but that the glycan-free trimer base contains accessible lysine residues, more so for the B41 genotype than the BG505 genotype (see Fig. A2). To make the coupling process more consistent and predictable, we designed C-terminal lysine-rich tags that could serve as a preferential attachment point at the SOSIP trimer base. After conducting pilot production and IO-NP coupling experiments using BG505 and/or B41 SOSIP.v4.1 trimers and various tag designs, we chose the KGKGKGK tag (designated KG4) for routine use. The KG4 tag did not adversely affect the trimer structure, as judged by NS-EM imaging (see Fig. A3A). Modeling showed that the three KG4 tags are prominent features of the B41 SOSIP.v4.1 trimer base; all four lysine residues (K665, K667, K669, K671) are accessible, although the endogenous K34 and K502 residues are now obscured by the tag (Fig. 3; see Fig. A2A). The BG505 SOSIP.v4.1-KG4 trimer attached to IO-NPs at a stoichiometry of 18 to 20 trimers per particle, higher than the 7 to 9 value for the nontagged version and implying that the tags were working as intended (Table 1). An ELISA showed that the VRC01, PGT145, and 10A NAb epitopes were retained, while the RM20A2 and RM19B1 trimer base non-NAb epitopes were substantially occluded (see Fig. A3B). The KG4-tagged trimers from the B41, 16055, and CZA97 genotypes were also successfully coupled to IO-NPs in appropriate orientations, again, as judged by ELISA (Table 1; see Fig. A3C to E).

In summary, the KG4 tag should facilitate attachment of any trimer genotype, particularly those lacking the lysines present at B41 positions K33 and K34 (e.g., BG505, where these residues are N33 and L34, respectively). For some genotypes, it is possible that mutating other exposed surface lysines or blocking them via knocked-in glycans may be beneficial for preventing attachment in an unwanted orientation.

### Antigenicity of B41 SOSIP.v4.1-KG4 IO-NP trimers by NAb depletion assay.

**FIG 4:**
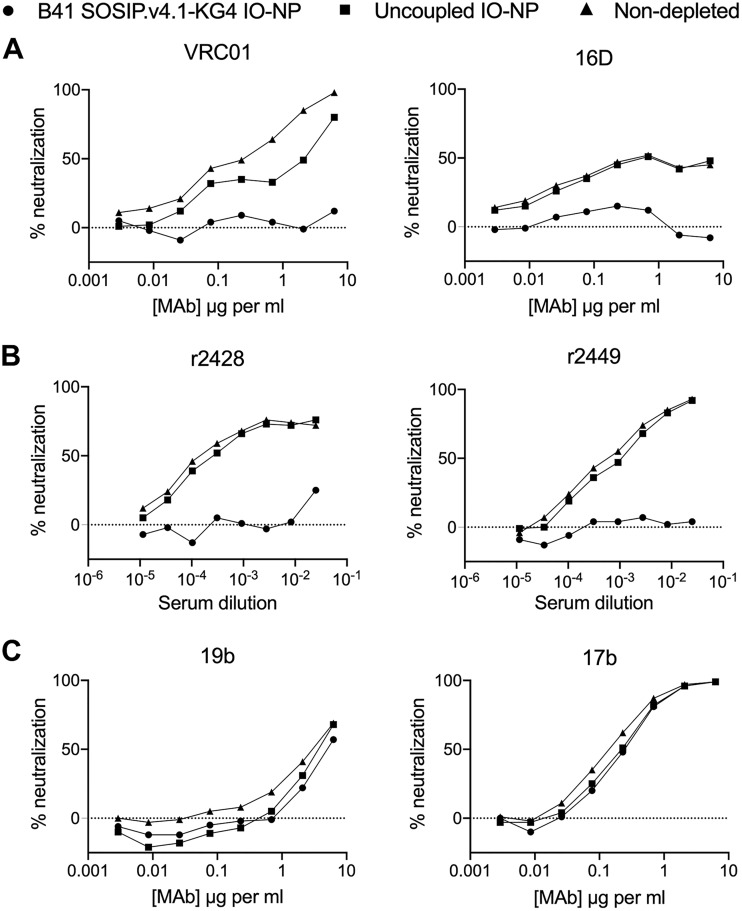
Antigenicity of B41 SOSIP.v4.1-KG4 IO-NP trimers by NAb depletion assay. (A) bNAb VRC01 or rabbit MAb 16D was incubated without (▲) or with (●) B41 SOSIP.v4.1-KG4 IO-NP trimers or with control, uncoupled IO-NPs (■), before assessing B41 virus neutralization. (B) As in panel A, except that rabbit anti-B41 SOSIP.v4.1 trimer serum samples r2428 and r2449 were used. (C) As in panel A, except that the MAbs were 17b and 19b and the test virus was MW965.26. This experiment was performed at DUMC.

**FIG A4:**
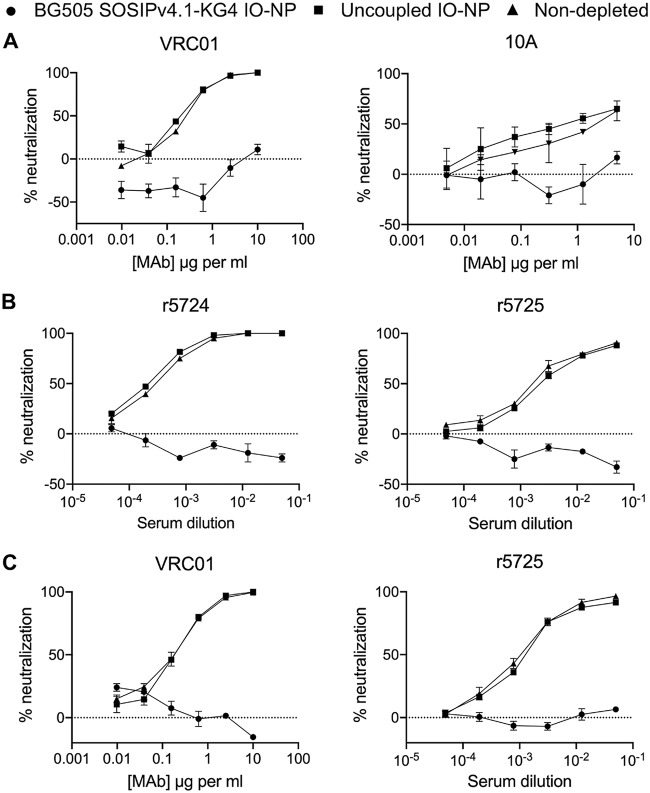
Antigenicity of BG505 SOSIP.v4.1-KG4 IO-NP trimers by an NAb depletion assay. (A) bNAb VRC01 or rabbit MAb 10A was incubated without (▲) or with (●) BG505 SOSIP.v4.1-KG4 trimer IO-NPs or with control, uncoupled IO-NPs (■) before testing for BG505.T332N virus neutralization. (B) As in panel A, except that rabbit anti-BG505 SOSIP.664 trimer serum samples r5724 and r5725 were used. (C) As in panel A, except that bNAb VRC01 and rabbit serum sample r5725 was used and the test virus was MW965.26. This experiment was performed at WCMC.

ELISAs can yield artefactual information on the exposure of V3 non-NAb epitopes on SOSIP trimers (46–48). As the magnetic properties of IO-NPs compromised solution-phase methods, such as surface plasmon resonance and biolayer interferometry, we used an NAb depletion assay to further study B41 SOSIP.v4.1-KG4 IO-NP trimers. The trimer-bearing and uncoupled, control IO-NPs were incubated in solution for 1 h with bNAb VRC01 or the neutralizing N289 glycan hole MAb 16D. After magnetically pelleting the IO-NPs, the supernatants were tested for B41 virus neutralization (Fig. 4A). The two NAbs were substantially depleted by the trimer IO-NPs; the control IO-NPs modestly depleted VRC01, probably because of nonspecific absorption of the bNAb to their lipid coating. Autologous NAbs present in sera from two rabbits immunized four times with B41 SOSIP.v4.1 trimers were also efficiently depleted by IO-NP trimers (Fig. 4B). In contrast, there was no reduction in neutralization of the tier 1 virus MW965.26 when the same IO-NP trimers were incubated with MAbs 19b (V3 specific) and 17b (CD4i specific) (Fig. 4C). Thus, B41 SOSIP.v4.1 IO-NP trimers present NAb epitopes under solution-phase conditions, while the V3 non-NAb 19b epitope is exposed only under ELISA conditions (Fig. 1C versus Fig. 4C) (50). In a similar experiment, BG505 SOSIP.v4.1-KG4 IO-NP trimers depleted the neutralizing activities of the VRC01 bNAb, the rabbit MAb 10A, and two serum samples from BG505 SOSIP.664 trimer-immunized rabbits (see Fig. A4A and B). In summary, the solution-phase NAb depletion assay confirmed that trimers attached to the IO-NPs had appropriate antigenicity.

### Bivalent IO-NPs carrying B41 and BG505 SOSIP.v4.1-KG4 trimers.

**FIG 5:**
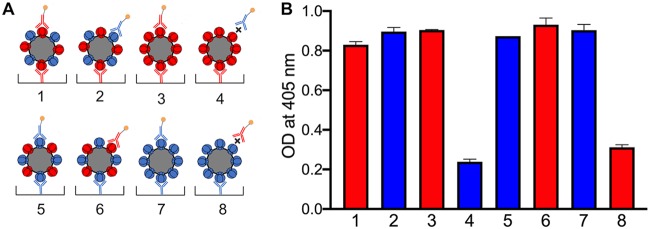
Validation of bivalent trimer IO-NPs by ELISA. (A) Schematic of monovalent and bivalent IO-NPs bearing BG505 (red) and/or B41 (blue) SOSIP.v4.1-KG4 trimers captured and/or probed with BG505-specific MAb 10A (red) or B41-specific MAb 16D (blue) at 5 μg/ml. The X symbol denotes nonreactivity between the MAb and the trimer. (B) Binding of MAb 10A (red) or 16D (blue) to the captured monovalent or bivalent IO-NP trimers under the conditions shown in panel A. The low-level signals for the two MAb-trimer mismatches (X in panel A) reflect the background binding of the MAbs to the IO-NP surface.

Presenting two different influenza virus hemagglutinin (HA) variants on the same ferritin NP is beneficial for inducing neutralization breadth in mice (19). As a proof of concept, we made a bivalent BG505 plus B41 SOSIP.v4.1-KG4 IO-NPs by adding the two soluble trimers in equimolar amounts to a coupling reaction mixture. Each monovalent IO-NP trimer was also prepared. The various particles were evaluated by ELISAs based on neutralizing anti-glycan hole MAbs specific for one of the two trimer genotypes. When an IO-NP is captured via an MAb to one trimer genotype (e.g., 10A to BG505) and then recognized by a biotin-labeled MAb to the second genotype (e.g., 16D to B41), both trimers must be present on the same particle, as was observed (Fig. 5). The total number of trimers on the bivalent IO-NPs was estimated to be 18 to 20, comparable to that for each monovalent IO-NP (Table 1). The similar optical density (OD) values obtained with the various trimer-MAb combinations suggest that the BG505 and B41 trimers are each present in comparable amounts (i.e., ∼9 to 10 of each) on the bivalent particles.

### Immunogenicity of B41 SOSIP trimer IO-NPs and/or B41 SOSIP trimer IO-NPs with TCHE tags.

**FIG 6:**
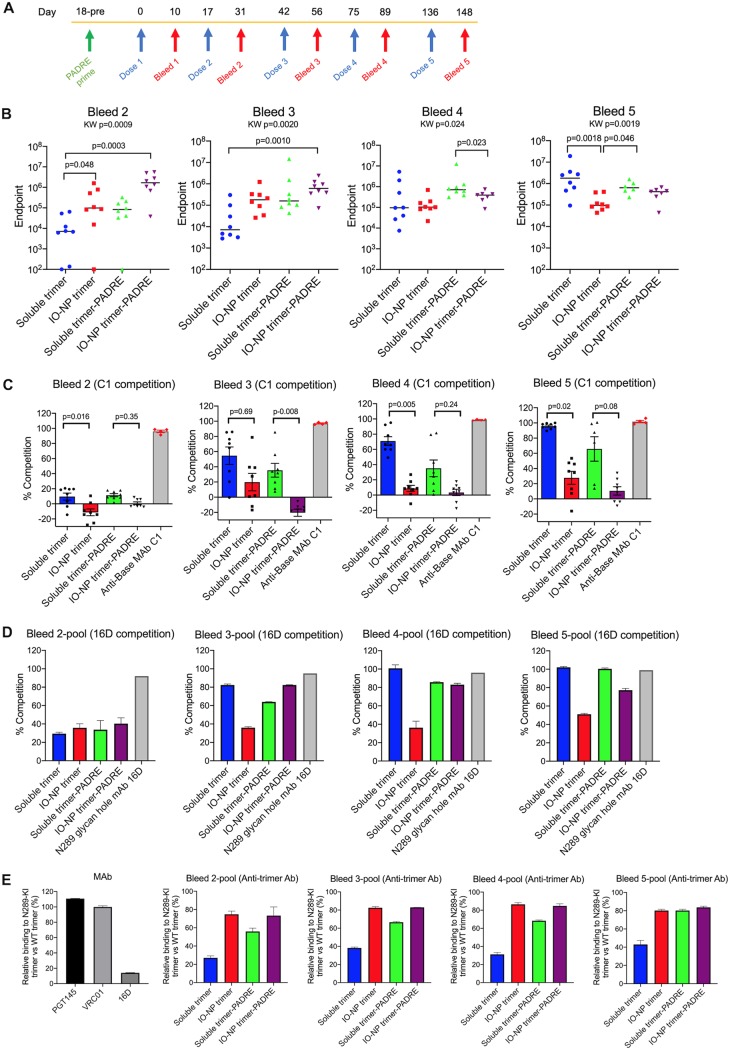
Antibody responses in B41 SOSIP.v4.1 trimer-immunized mice. (A) Schematic immunization protocol showing the times of the five soluble or IO-NP trimer doses (blue arrows) and the subsequent bleeds (red arrows). All mice were primed with the PADRE peptide 2 weeks before the first trimer immunization (green arrow). Group designations are as follows: 1, mice immunized with soluble trimer; 2, mice immunized with IO-NP trimer; 3, mice immunized with soluble trimer-PADRE-v3; 4, mice immunized with IO-NP trimer-PADRE-v3. KW, Kruskal-Wallis test. (B) Anti-trimer Ab ELISA titers in individual mice for bleeds 2 to 5 (the bars indicate the medians for each group, and the results for individual mice are shown; each data point is the mean from three replicates). (C) Anti-base Ab responses in the same sera (1:100 dilution) were determined by a competition ELISA using biotin-labeled rabbit MAb C1. The data plotted represent the percent inhibition of C1 binding by sera at a dilution of 1/100. In the same assay, unlabeled C1 (1 μg/ml) gave 100% competition. (D) Ab responses to the N289 glycan hole in pools of sera from bleeds 2 to 5 (1:100 dilution), as determined by a competition ELISA using biotin-labeled rabbit MAb 16D. The data plotted represent the percent inhibition of 16D binding by sera at a dilution of 1/100. In the same assay, unlabeled 16D (1 μg/ml) gave 90 to 100% competition. (E) On the left is shown the percent binding of MAbs PGT145, VRC01, and 16D at 10 μg/ml to B41 SOSIP.v4.1-N289-KI trimers relative to that to wild-type B41 SOSIP.v4.1 trimers. In the other four charts, serum pools from mouse bleeds 1 to 4, as indicated, were assessed for anti-trimer antibodies in the same way. The data plotted represent the proportions of anti-trimer Abs that bound to the N289-KI trimer compared to wild-type (WT) trimers at a serum dilution of 1/100 (defined as 100%).

**FIG A5:**
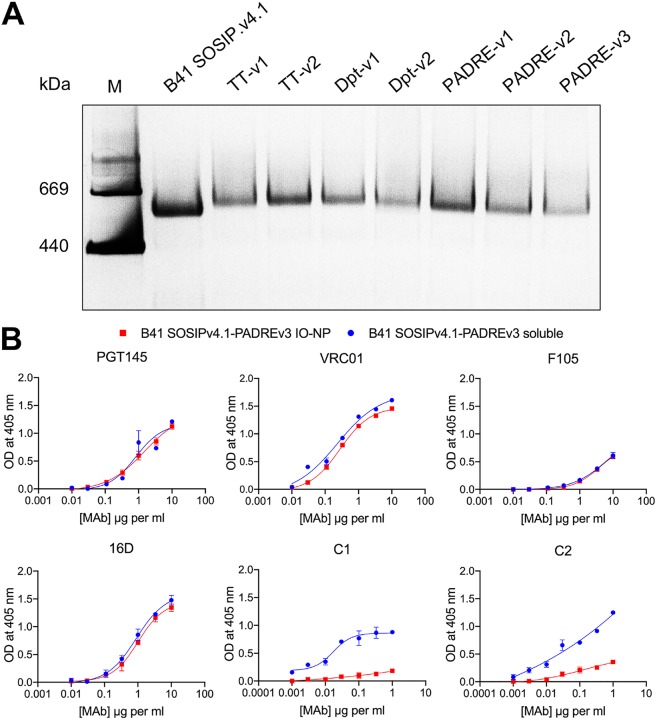
C-terminal TCHE tags on B41 SOSIP.v4.1 trimers. (A) BN-PAGE analysis of the trimer variants shown, showing a single band with a molecular weight consistent with trimer formation. Lane M, marker proteins. See also Fig. S1A. (B) Results of a 2G12-capture ELISA of B41 SOSIP.v4.1-PADRE-v3 soluble (blue symbols) and IO-NP (red symbols) trimers. The bNAbs PGT145 and VRC01, non-NAb F105, and rabbit MAbs 16D, C1, and C2 were tested, as indicated.

**FIG A6:**
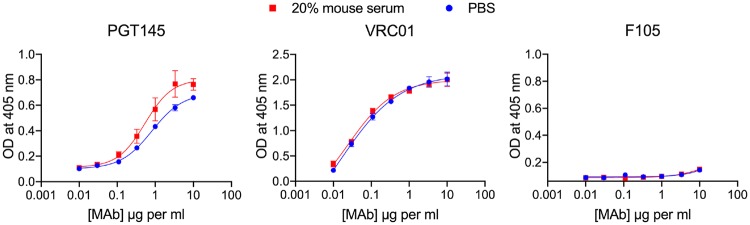
Stability of B41 SOSIP.v4.1 IO-NP trimers in mouse serum. The IO-NP trimers were incubated overnight at 37°C in PBS with (red symbols) or without (blue symbols) 20% normal mouse serum and then washed and assessed for antigenicity by a 2G12-capture ELISA using bNAbs PGT145 and VRC01 and non-NAb F105, as indicated.

Our goal in an initial immunogenicity study was to compare soluble and IO-NP trimers that did or not bear a C-terminal TCHE tag (Fig. 6). We evaluated various TCHE tag constructs for yield and trimer formation and chose the B41 SOSIP.v4.1-PADRE-v3 design (Table 1 and Table 2; see Fig. A5A and B). Env trimer-liposomes are reportedly unstable in animal serum (25, 51). Before testing B41 SOSIP.v4.1 IO-NP trimers in mice, we assessed their stability in mouse serum and found no evidence for instability, as judged by retention of the PGT145 and VRC01 epitopes on the attached trimers (see Fig. A6).

**TABLE 2:**
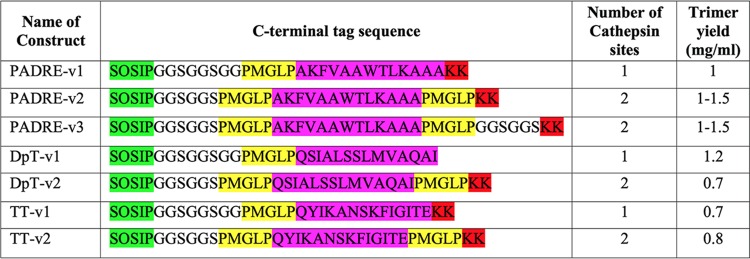
Design of C-terminal TCHE tags on B41 SOSIP.v4.1 trimers^*a*^

a TT, tetanus toxoid; DpT, diphtheria toxoid. The GS-rich spacer is in black, cathepsin S cleavage sites are highlighted in yellow, TCHE sequences are in magenta, and additional lysine residues are in red. Cathepsin S is a lysosomal protease active in the pH range from 6 to 7.5; the cleavage site is intended to allow the TCHE sequence to be liberated from the trimer and, by extension, the trimer IO-NP. The KK motif was added as an early strategy, not further pursued, to provide a preferential point of trimer attachment to IO-NPs.

**FIG A7:**
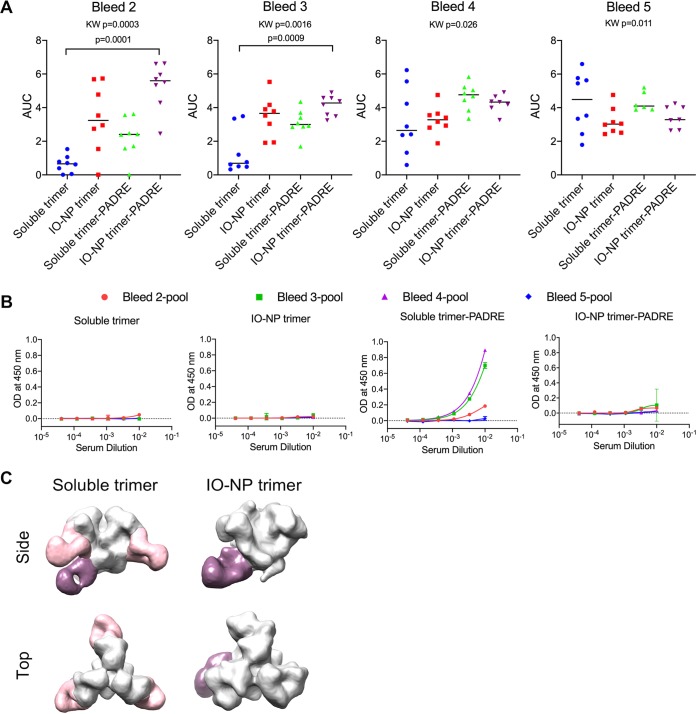
Immunogenicity of B41 SOSIP.v4.1 soluble and IO-NP trimers in mice. (A) AUC analysis of the anti-trimer binding Ab ELISA data presented as endpoint titers in Fig. 6B. (B) The immunogenicity of the PADRE tag on B41 SOSIP.v4.1 trimers was assessed by ELISA using a His-tagged version of the PADRE peptide. Pools of sera from bleeds 2 to 5 were tested. Red, bleed 2; green, bleed 3; purple, bleed 4; blue, bleed 5. (C) Negative-stain EM 3D reconstructions of complexes between B41 SOSIP.v4.1 trimers and Fabs derived from group 1 and group 2 sera, showing N289 glycan hole-like antibodies (pink) and base-binding antibodies (purple). We were unable to test Fabs from group 3 and 4 sera with the B41 SOSIP.v4.1-PADRE-v3 immunogen due to limited sample availability.

Four groups of mice (*n* = 8) were primed with a PADRE peptide 25 days before the first of five trimer immunizations, given intraperitoneally (i.p.) over an ∼21-week period (Fig. 6A). The mice were bled 10 to 14 days after each immunization. Serum antibody endpoint titers were determined by ELISA using a His-tagged B41 SOSIP.v4.1 trimer. The anti-trimer antibody titers after trimer dose 1 were very low in each group (Table 3). The highest titers after the second and third doses were seen in mice given the IO-NP trimer-PADRE immunogen (group 4), and the lowest were seen in the soluble trimer recipients (group 1), with intermediate titers being seen in the other two groups (Fig. 6B; Table 3). An area-under-the-curve (AUC) analysis yielded similar differentials (see Fig. A7A). After the second trimer dose, the median endpoint titer of 1,700,000 in group 4 mice (IO-NP trimer-PADRE) was ∼240-fold higher than that in group 1 mice (soluble trimer) (Fig. 6B; Table 3). The titer differential between the groups was sustained after the third dose but diminished after the fourth and fifth doses; the highest median titer, 1,800,000, after dose 5 was in group 1 (soluble trimer), and the lowest, 97,000, was in group 2 (IO-NP trimer) (Fig. 6B; Table 3). Thus, for anti-trimer antibody titers, IO-NP presentation and the TCHE tag each provided a substantial initial immunogenicity benefit that was not sustained. In contrast, the median titers in both soluble trimer groups increased after each immunization; after the fifth dose, they approximated those seen in group 4 (IO-NP trimer-PADRE) after three doses (Fig. 6B; Table 3).

**TABLE 3:** Anti-B41 SOSIP.v4.1 trimer binding antibody endpoint titers in mouse sera^*a*^

Group	Immunogen	Titer				
		Day 10, bleed 1	Day 31, bleed 2	Day 56, bleed 3	Day 89, bleed 4	Day 148, bleed 5
1	Soluble trimer	<100	7,100	7,100	96,000	1,800,000
2	IO-NP trimer	100	98,000	180,000	100,000	97,000
3	Soluble trimer-PADRE	<100	83,000	160,000	710,000	640,000
4	IO-NP trimer-PADRE	280	1,700,000	610,000	390,000	420,000

a ELISA endpoint titers were determined against the His-tagged version of the B41 SOSIP.v4.1 soluble trimer immunogen; the values shown are the median values for each immunization group.

To assess anti-base non-NAb responses, we used a competition ELISA based on serum inhibition of MAb C1 binding (Fig. 6C). The low antigenicity of the base on IO-NP trimers (Fig. 1D; see Fig. A5B) was reflected by the low serum anti-base responses at all time points for the two IO-NP trimer groups. Thus, trimer base responses (i.e., Abs able to inhibit C1 binding) generally increased in both soluble trimer groups over time but were always weaker in the two IO-NP trimer groups (groups 2 and 4 versus groups 1 and 3) (Fig. 6C). Antibodies to the PADRE tag were seen in group 3 (soluble trimer-PADRE) but not in group 4 (IO-NP trimer-PADRE), implying that the tag is poorly accessible at the interface between the trimers and the particle surface (see Fig. A7B).

Another competition ELISA, based on NAb 16D, was used to probe for serum Abs to the single autologous NAb epitope, the N289 glycan hole, presented by B41 SOSIP trimers and the corresponding virus (52). After the second immunization, low and comparable levels of 16D-competing Abs were seen in serum pools from all four groups (Fig. 6D). The extent of competition then increased after further immunizations in groups 1, 3 (soluble trimers), and 4 (IO-NP trimer-PADRE) but not in group 2 (IO-NP trimer) (Fig. 6D). We used the same serum pools to compare anti-trimer Ab responses measured using the wild-type B41 SOSIP.v4.1 trimer and its N289-knock-in (N289-KI) variant (Fig. 6E). The VRC01 and PGT145 bNAbs bound comparably to the two trimers, but NAb 16D to the N289 glycan hole recognized only the wild-type version. Serum Ab titers against the N289-KI trimer were always lower than those against the wild type, but the differential was generally greater for groups 1 and 3 (soluble trimers) than for groups 2 and 4 (IO-NP trimers) (Fig. 6E).

We used NS-EM to image complexes between the B41 SOSIP.v4.1 trimer and Fabs purified from terminal bleed (day 189) sera for groups 1 and 2 (53). Fabs to the N289 glycan hole epitope and the trimer base were visible in group 1 samples (soluble trimer), but only anti-base Fabs were visible in group 2 samples (IO-NP trimer) (see Fig. A7C). Hence, by this assay also, the autologous NAb epitope was not immunogenic on IO-NP trimers. Taken together, the various assays show that, compared to soluble trimers, a substantially smaller component of the overall Ab response induced by IO-NP trimers is directed against the N289 glycan hole NAb epitope (Fig. 6D and E; see Fig. A7C). The electron microscopy (EM) assay does not quantify the amount of anti-base Abs but shows that some are induced by IO-NP trimers, despite the reduced antigenicity and immunogenicity of this region, as probed using MAbs (Fig. 1D and 6C).

**FIG 7:**
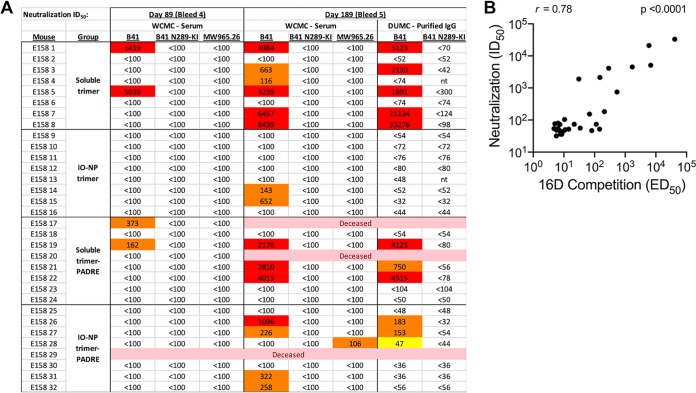
Neutralization of B41 virus by sera from soluble and IO-NP trimer-immunized mice. (A) Data are shown for individual mice in two repeat experiments, as indicated, after immunizations 4 and 5 (no sera from earlier time points had NAb titers of >100; not plotted). Mice that died during the experiment are indicated. The N289-KI mutation closes the N289 glycan hole epitope for autologous NAbs. MW965.26 is a heterologous tier 1A virus. The data in columns marked WCMC were generated at Weill Cornell Medical College using sera; the data in columns marked DUMC (bleed 5 only) were from Duke University Medical Center (DUMC) and were generated using purified IgG. (B) Correlation between the midpoint titers (50% effective dose [ED_50_]) of antibodies able to inhibit the binding of NAb 16D to its N289 glycan hole epitope on B41 SOSIP.v4.1 trimers (Fig. 6D, but in which data are for individual sera and not pools) and the B41 neutralization titers (50% inhibitory dose [ID_50_]) derived using the same sera (Fig. 7A). A nonparametric Spearman correlation gave an *R* value of 0.78 (*P* < 0.0001).

We tested mouse sera for B41 virus neutralization in Tzm-bl cell assays. No NAbs were detected after the first three immunizations, but modest to strong neutralization was seen with 15 out of 32 serum samples from the last two bleeds (Fig. 7A). IgG Abs purified from bleed 5 sera were assessed independently, with comparable outcomes (Fig. 7A). Higher NAb titers were induced by soluble trimers than by IO-NP trimers (IgG titers; *P* = 0.006). The NAbs were specific to the N289 glycan hole, as the B41 N289-KI virus mutant was resistant (Fig. 7A). The MW965.26 virus was not neutralized by any sera, showing that V3-targeting or other NAbs to tier 1A strains were not induced by B41 SOSIP.v4.1 immunogens (see also Fig. A7C). There was a very strong correlation between the endpoints of the N289 glycan hole competition ELISA and the B41 virus neutralization assay (Fig. 7B).

We conclude that autologous NAbs against the N289 glycan hole are induced in mice by soluble B41 SOSIP.v4.1 trimers but are only inefficiently induced by the corresponding IO-NP trimers.

### Recombinant and serum MBL binding to soluble and IO-NP SOSIP trimers.

**FIG 8:**
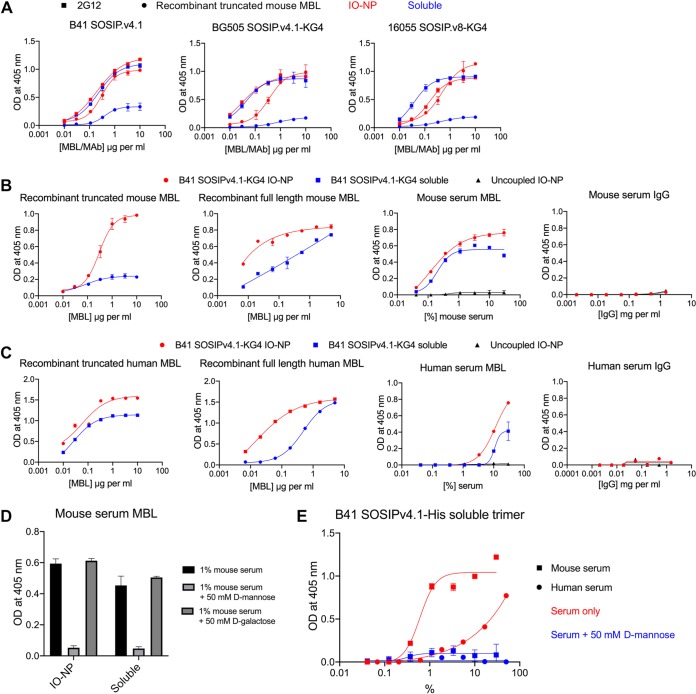
MBL reactivity with soluble and IO-NP trimers. (A) B41 SOSIP.v4.1 (nontagged), BG505 SOSIP.v4.1-KG4, or 16055 SOSIP.v8-KG4 soluble (blue) or IO-NP (red) trimers (150 ng of Env) were captured onto ELISA plate wells via bNAb VRC01. Mouse recombinant truncated MBL (circles) or biotin-labeled bNAb 2G12 (squares) was then titrated and detected. In this and related panels, 2G12 binding served to verify that the Env contents of the captured soluble and IO-NP trimers were similar. (B) VRC01-captured B41 SOSIPv4.1-KG4 soluble and IO-NP trimers were incubated with a range of concentrations of recombinant truncated or full-length mouse MBL, normal mouse serum, or mouse serum IgG, as indicated. Uncoupled (i.e., no trimers attached) IO-NPs were also tested to control for nonspecific binding of serum MBL. Trimer-bound murine MBL was detected. (C) As for panel B, except that the B41 SOSIPv4.1-KG4 soluble and IO-NP trimers were incubated with recombinant truncated or full-length human MBL, normal human serum, or human serum IgG. Uncoupled (i.e., no trimers attached) IO-NPs were also tested to control for nonspecific binding of serum MBL. Trimer-bound human MBL was detected. (D) Mouse serum (1% by volume) was incubated with 50 mM d-mannose or d-galactose, before addition to VRC01-captured B41 SOSIPv4.1-KG4 soluble or IO-NP trimers. Trimer-bound murine MBL was detected. (E) Soluble B41 SOSIP.v4.1-His trimers were captured onto Ni-NTA ELISA plate wells via their His tags. Mouse or human serum was titrated in the presence or absence of 50 mM mannose, as indicated, and trimer-bound MBL was detected.

MBL binding to trimer-NPs, but not soluble trimers, can trigger distinctive, MBL- and complement-dependent antigen trafficking to follicular dendritic cells and germinal centers in mice (15). Assessed by a VRC01-capture ELISA, murine recombinant MBL (truncated or full length) bound much more strongly to B41 (KG4 tag or not), BG505, and 16055 SOSIP IO-NP trimers than to the corresponding soluble trimers (Fig. 8A and B). Under the same conditions, the glycan-reactive 2G12 bNAb bound similarly to the B41, BG505, and 16055 soluble and IO-NP trimers (Fig. 8A). The MBLs present in normal, non-heat-inactivated mouse serum also bound strongly to the B41 IO-NP trimers, but, unlike the recombinant MBLs, they bound comparably to the B41 soluble trimers. Purified mouse IgG was, in contrast, nonreactive (Fig. 8B). Similar findings were made using recombinant truncated and full-length human MBLs, although the differential reactivity with the IO-NP trimers versus soluble trimers was markedly less for the truncated human MBL than for its murine counterpart (Fig. 8C; cf. Fig. 8B). Human serum MBL, but not purified IgG, was also trimer reactive (Fig. 8C). The ∼10-fold higher serum concentrations needed to see human MBL reactivity than murine MBL reactivity are consistent with the reported MBL contents of the sera of the two species, i.e., 1 to 2 μg/ml for human serum versus 25 to 90 μg/ml for mouse serum (30 μg/ml for serum from the C57BL/6J strain used in the immunization experiments) (54, 55). The binding of the murine and human serum MBLs to the IO-NPs was trimer dependent, as no reactivity was seen using uncoupled IO-NPs (Fig. 8B and C). Furthermore, mouse serum MBL reactivity with B41 soluble and IO-NP trimers was inhibited by d-mannose but not by d-galactose (Fig. 8D). These two sugars are and are not competitors of MBL-ligand interactions, respectively (56). Finally, to guard against the possibility that MBL reactivity with the soluble trimers was an artifact of using the VRC01 bNAb as an ELISA capture reagent, we showed that mouse and human serum MBLs bound in a mannose-sensitive manner to B41 SOSIP.v4.1-His trimers that had been captured onto Ni-nitrilotriacetic acid (NTA) wells via the His tag (Fig. 8E). The recombinant MBLs could not be studied in this assay, as they have His tags and, hence, bind directly to the ELISA wells.

### MBL binding IO-NP trimers impairs access to the autologous NAb epitope and some bNAb epitopes.

**FIG 9:**
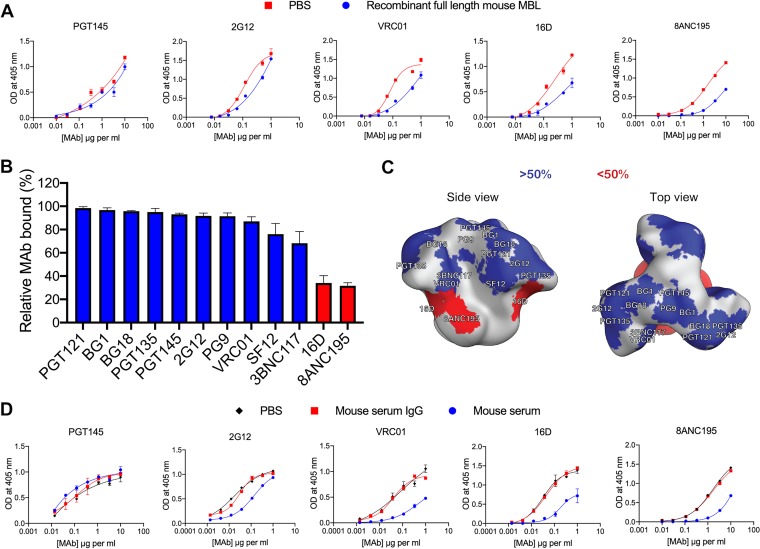
MBL inhibition of NAb binding to IO-NP SOSIP trimers. (A) VRC01-captured B41 SOSIP.v4.1-KG4 IO-NP trimers were exposed to recombinant full-length mouse MBL (5-μg/ml final concentration) before addition of the indicated anti-trimer MAbs at a range of concentrations. (B) The bar chart depicts the percent binding of various anti-trimer MAbs to VRC01-immobilized B41 SOSIP.v4.1-KG4 IO-NP trimers in the presence of full-length recombinant mouse MBL (5-μg/ml final concentration) relative to that with no MBL addition (100% binding). The MAb concentrations were based on earlier titration curves and were intended to generate OD values in the range of 0.7 to 1.0, a signal level that approaches the maximum extent of MAb binding under the assay conditions, and were as follows: BG18, PGT135, SF12, and BG1, 3.3 μg/ml; PGT145, PGT121, 8ANC195, VRC01, 3BNC117, and PG9, 1 μg/ml; and 16D and 2G12, 0.3 μg/ml. Citations describing the MAbs used and their epitopes are located in the Materials and Methods section. The bars are ranked from the highest to the lowest relative binding (in percent) and represent the means from 2 replicate ELISAs + SEM. The red bars indicate that inhibition was >50%. (C) The epitopes for the various MAbs tested in the assay whose results are presented in panel B are plotted on the B41 SOSIP trimer surface. The red patches indicate <50% MAb binding in the presence of MBL at the concentrations tested (see the legend to panel B). (D) As for panel A, except that the B41 SOSIP.v4.1-KG4 IO-NP trimers were exposed to PBS, normal mouse serum (10% by volume, i.e., a 1:10 dilution), or normal mouse IgG (200 μg/ml) before addition of the anti-trimer MAbs.

We assessed the impact of recombinant full-length murine MBL on the reactivity of various anti-trimer MAbs with B41 SOSIP.v4.1-KG4 IO-NP trimers (Fig. 9). This MBL had only a limited impact on the binding of the PGT145 and 2G12 bNAbs but substantially inhibited the autologous 16D N289 glycan hole autologous NAb and the 8ANC195 bNAb and impeded VRC01 binding at some input concentrations (Fig. 9A). Testing a larger panel of bNAbs identified 8ANC195 as being particularly susceptible to MBL inhibition, whereas others were unaffected or only minimally so at the input concentrations tested (Fig. 9B). A depiction of the NAb and bNAb epitope footprints on the trimer surface suggests that recombinant mouse MBL binds near the base of the trimer in a way that affects the closely proximal 16D and 8ANC195 epitopes (Fig. 9C). Note, however, that the extent of inhibition is dependent on the input concentration of both the MAb and the MBL. Thus, under some concentration conditions, VRC01 binding was quite sensitive to the presence of MBL (see, for example, Fig. 9A), suggesting that the MBL footprint may extend further up the trimer than indicated in Fig. 9C. Mouse serum strongly inhibited 16D binding to the same IO-NP trimers, but also had substantial blocking effects on the 2G12, VRC01, and 8ANC195 bNAbs, while it did not affect PGT145 binding. In each case, a comparable concentration of purified mouse IgG was not inhibitory (Fig. 9D). The inhibition pattern suggests that the (native) MBL footprint could affect some bNAb epitopes located in the low and central regions of the IO-NP-attached trimer but not ones located at the apex.

We conclude that recombinant and native (serum) MBLs can bind to IO-NP trimers and, in some cases, soluble trimers in a way that can impede access to NAb or bNAb epitopes to various degrees. As the immunogenicity of the affected epitopes could be affected, additional studies on MBL binding to trimer-bearing NPs and the soluble trimer are required to better understand the implications.

## DISCUSSION

We sought to increase vaccine-elicited responses to HIV-1 Env by presenting SOSIP trimers as particulate antigens and/or by adding a C-terminal TCHE. Commercially available IO-NPs are used safely in humans (31–33). The straightforward chemistry for linking SOSIP trimers to IO-NPs is performed under benign pH and temperature conditions. Interprotomer amine-carboxyl cross-linking via similar glutaraldehyde or 1-ethyl-3-(3-dimethylaminopropyl)carbodiimide (EDC)/*N*-hydroxysuccinimide (NHS) chemistry does not compromise SOSIP trimer antigenicity or immunogenicity (57, 58). The magnetism of the iron oxide core facilitates IO-NP trimer production and usage, e.g., in NAb depletion assays.

We first made SOSIP trimer IO-NPs based on the BG505 (clade A) and B41 (clade B) virus genotypes. The orientation of SOSIP trimers on IO-NPs is not fully controlled because the attachment chemistry involves reacting the cross-linker with lysine residues exposed on the trimer surface. Variation in the numbers, locations, and accessibilities of lysine residues between trimer genotypes and/or subdesigns could impose a random component on the attachment process. However, modeling of the B41 SOSIP.v4.1 trimer shows that glycans occlude most surface lysines, except for those on the base, the predominant region where these trimers attach to IO-NPs, according to NAb and non-NAb binding data. In contrast, several lysines are exposed as a patch on the side of the BG505 SOSIP trimer. Antigenicity profiles indicate that BG505 SOSIP.664 trimers attach to IO-NPs in an ill-defined orientation that may involve lysines on the side of the trimer and/or near its apex. However, their V3-stabilized BG505 SOSIP.v4.1 counterparts attach predominantly via the base, although at a lower stoichiometry.

The efficiency, orientation, and generality of coupling to IO-NPs benefit when a C-terminal lysine-rich tag (KG4) on the trimer base creates a preferential attachment point. In this orientation, otherwise highly immunogenic and possibly distractive non-NAb neoepitopes on the trimer base are less accessible. Via KG4 tags, we made monovalent CZA97 and 16055 clade C trimer IO-NPs and bivalent ones displaying both B41 and BG505 SOSIP.v4.1 trimers. A bivalent (or multivalent) trimer IO-NP may be useful; presenting two different influenza virus HA variants on the same ferritin NP induced a better neutralization breadth in mice than a 1:1 mixture of the two individual HA-NPs or either monovalent one (19). In principle, many different SOSIP-KG4 trimers could be attached to the same IO-NP, although in practice each would need to be present in a relevant amount.

NAb depletion assays showed that B41 and BG505 SOSIP.v4.1 trimers had appropriate antigenicity properties after IO-NP attachment when tested under non-ELISA conditions. Furthermore, NAbs induced in rabbits by B41 and BG505 SOSIP trimers were removed from sera by the corresponding IO-NP trimers. Showing that NAbs are Env specific and mapping their epitopes have involved incubating soluble Env proteins with serum and virus and then adding the mixture to target cells (59). Env mutants with a disabled CD4bs must, however, be used because gp120 binding to cell surface CD4 interferes with HIV-1 infection and the NAb assay endpoint; a new Env protein must be produced, and NAbs to the CD4bs cannot be analyzed (59). In contrast, IO-NPs bearing SOSIP trimers with a wild-type CD4bs can be used to map NAb responses because the particles and trimer-bound NAbs are magnetically removed from the virus-serum mixture and never come into contact with cell surface CD4.

We sought to attach ∼15 to 20 trimers per IO-NP, on average, because the similarly sized I53-50 self-assembling protein NP bears 20 trimers (26, 27). This stoichiometry range was achieved with B41 SOSIP.v4.1 trimers and the BG505 SOSIP.v4.1-KG4 trimers. The B41 SOSIP.v4.1 IO-NP trimers triggered Ca^2+^ signals from VRC01 BCR-expressing B cells more strongly than the same soluble trimers, as seen with other NP designs (15, 26, 60). The density of epitopes on NPs influences BCR cross-linking and B-cell activation (13, 61, 62). In a virus-like particle-based study, the magnitude of Ab responses in mice was correlated with the epitope density, with 60 epitopes spaced 5 to 10 nm apart being optimal (63). Dense packing of SOSIP trimers on liposomes was also beneficial in mice (25). If larger (e.g., 50-nm-diameter) IO-NPs become available, they could present a greater number of the same or different SOSIP trimers. Also, NPs of ∼20 to 30 nm traffic to lymph nodes within 2 h without the involvement of migratory antigen-presenting cells (APCs), while larger ones linger longer at the injection site until APCs arrive (13, 64, 65). NPs larger than 100 nm may be excluded from lymph nodes (13, 15).

We made B41 SOSIP.v4.1 trimers with C-terminal TCHEs to try to overcome any limitations to Env immunogenicity caused by suboptimal T-cell helpers (TCH). In mice and rabbits, a PADRE tag increased anti-gp120 and tier 1 NAb responses to gp120 monomers (66). Here, we evaluated B41 trimers with and without the PADRE-v3 TCHE tag and in soluble and IO-NP forms; all of the mice were primed for 25 days with the soluble PADRE peptide prior to the first trimer immunization. The TCHE tag and IO-NP presentation independently increased the anti-trimer Ab titers after the first two immunizations, and the endpoint titer in the IO-NP trimer-PADRE-v3 groups exceeding 1,000,000. However, the differences among the four groups diminished after additional doses, and the titers were fairly similar at the end of the study. The gradual development of *de novo* T-cell responses against Env epitopes that provide additional TCH may contribute to the equalization of the serum titers after multiple boosts. In future, we could use a dimeric TCHE memory peptide (TpD) containing both tetanus toxoid and diphtheria toxoid TCHEs linked by a cathepsin site, which strongly benefited the Ab responses to a nicotine vaccine in mice and macaques; and all 20 normal human blood donors generated a T-helper-cell recall response to the TpD peptide (67). Intrastructural T-cell help (ISH) involves Gag or other non-Env TCHEs indirectly benefitting anti-Env Ab responses (68–73). We could exploit this mechanism by coupling a TCH peptide, such as PADRE and a SOSIP trimer(s), to IO-NPs to copresent TCH and bNAb epitopes that are not in the same polypeptide chain, noting that anti-V3 Ab titers were substantially higher when NPs copresented V3 and PADRE peptides than when the same two peptides were presented on separate NPs and given as a mixture (74).

In a serum competition ELISA, the B41 SOSIP.v4.1 trimer base was less immunogenic in the IO-NP context, consistent with antigenicity and modeling data showing that this region is partially occluded at the trimer-particle interface. More unexpected were competition ELISA data on the N289 glycan hole autologous NAb epitope. The extent of competition in this 16D NAb-based ELISA increased after each immunization in the two soluble trimer groups, but not in the IO-NP trimer group, and increased only partially when the PADRE tag was also present. Thus, N289 glycan hole-specific serum Ab responses were boosted in the soluble trimer groups but much less, or not at all, when the same trimers were presented on IO-NPs. EM imaging also found no evidence of anti-glycan hole Abs in the IO-NP trimer group. An ELISA comparing Ab responses to the immunogen trimer and a variant with the autologous NAb epitope closed via a N289 glycan-KI change further showed how poorly immunogenic this site was on IO-NP trimers compared to soluble trimers.

A strong autologous NAb response against the N289 glycan hole epitope on the B41 tier 2 virus was unexpected. Tier 2 NAbs have been rarely and only ever weakly elicited in mice given SOSIP or similar trimers (21, 43–45). The B41 NAb responses were almost exclusively seen in the soluble trimer groups, with only a single IO-NP trimer recipient responding, and were highly correlated with 16D NAb competition ELISA titers. As to why NAbs were induced in this study, the use of the B41 trimer genotype, the priming of the mice with a PADRE peptide, and the five trimer immunizations are all differences from prior protocols (21, 43–45). Additional studies are warranted to explore these variables.

Taken together, serology and neutralization assays clearly show that the sole autologous NAb epitope on the B41 trimers is poorly immunogenic when presented in the IO-NP context, although less so when the PADRE-TCHE tag was also present, yet this epitope was efficiently presented on the IO-NP trimers, judged by its accessibility to NAb 16D in both ELISA and NAb depletion assays. How can this context-dependent antigenicity versus immunogenicity discontinuity be explained? A clue comes from a report that BG505 SOSIP trimer ferritin NPs traffic in mice via an MBL-dependent antigen trafficking pathway that is not or that is much less available to the corresponding soluble trimers (15). We found that recombinant mouse MBLs reacted much more strongly with the B41 SOSIP.v4.1 IO-NP trimers than with the soluble trimers *in vitro* and that MBL binding interfered with binding of 16D to its N289 glycan hole epitope. However, MBLs present in mouse serum were more comparably reactive with the soluble and IO-NP trimers. The epitope occlusion effect of serum MBL binding may be sufficient to explain why IO-NP trimers were unable to induce autologous NAbs against the N289 glycan hole epitope *in vivo*. As the corresponding soluble trimers were also MBL reactive and did induce autologous NAbs, other factors might also be involved, such as the differential trafficking of soluble and NP trimers referred to above (15).

MBL binding to B41 IO-NP trimers also occluded bNAb epitopes to various extents, as did mouse serum, but purified IgG did not. Interpreting the bNAb inhibition pattern is not straightforward, because the epitope location, the binding affinity/kinetics of each bNAb, the biotin-labeling stoichiometry, and the MBL and bNAb concentrations tested can all affect the extent of inhibition seen in a competition ELISA. Moreover, there are multiple mannose moieties on the SOSIP trimer surface that could serve as MBL binding sites (75). Quantitative and qualitative differences between recombinant MBL and the mannose binding proteins present in mouse serum should also be considered. Nonetheless, our attempt to outline where recombinant, full-length murine MBL binds to IO-NP trimers pointed toward the lower half of the trimer as the likely site of interaction, with a possible extension toward the CD4bs (VRC01 epitope). Mouse serum MBLs strongly inhibited binding of the 16D autologous NAb and also affected bNAb epitopes further up the trimer (2G12, VRC01), although not the PGT145 site at the apex. In this context, a meta-analysis showed that, compared to soluble trimers, the most immunogenic sites on trimer-NPs in animal studies were located at or near the apex (16). Additional techniques are needed to further refine our understanding of where MBLs bind. As NPs of other designs interact with MBL *in vitro* and *in vivo*, epitope occlusion and other outcomes could affect how trimer-NPs in general are used for inducing particular bNAb classes (15). Species dependency should also be considered; for example, human serum may contain over 10-fold lower MBL concentrations than mouse serum (54, 55). Accordingly, we found that ∼10-fold higher human serum concentrations than mouse serum concentrations were required for binding to the B41 IO-NP trimers.

MBL binding may also influence the immunogenicity of soluble trimers and perhaps other forms of HIV-1 Env glycoproteins, which have long been known to be poor immunogens compared to other pathogen-relevant antigens (8, 9). Soluble gp120 monomers display multiple mannose moieties and SOSIP trimers do so even more, features that are not shared with other immunogens (75). The mannose content of HIV-1 Env proteins in general makes them targets for both MBL and membrane-anchored mannose binding C-type lectin receptors (MCLRs) (76). In comparative studies, soluble HIV-1 gp140 induced much greater IgG1/IgG2a/c ratios in mice than HIV-1 Gag, Influenza virus HA or RSV F proteins and the skewing effect of Env were imparted on the anti-Gag IgG response when an Env-Gag fusion protein was tested; this atypical IgG isotype response to HIV-1 gp140 may result from an MCLR interaction (77). Depleting or occluding mannose moieties on monomeric HIV-1 gp120s skewed the IgG isotype response of immunized mice away from IgG1 and toward IgG2a and IgG3 and increased the overall anti-gp120 titers (78, 79).

The full consequences of HIV-1 Env protein interactions with MBL or MCLRs are not yet clear, but the atypically high mannose content of the trimer, whether virion, NP, or soluble SOSIP, may be yet one more complex influence on the interplay between HIV-1 and the humoral immune system that is not shared with most other pathogens (8, 79–81). Accordingly, our new findings warrant further explorations both *in vitro* and *in vivo*, to better understand how innate immunity components could be suppressing antibody responses to HIV-1 Env proteins, particularly when they are presented on NPs of various designs. Our observations on MBL reactivity with IO-NP trimers and NAb epitope occlusion *in vitro* correlate with the outcomes of immunogenicity studies *in vivo*. Additional studies will, however, be required to establish causation. The generalization of our findings to other designs of trimer-NPs will also need to be assessed, although there are no grounds to believe that what we have seen is unique to the IO-NP method. If MBL binding does compromise the utility of NPs for presenting HIV-1 Env proteins, the flexibility offered by the IO-NP design may still be useful for presenting other, less-mannose-rich pathogen antigens as a particulate vaccine.

## MATERIALS AND METHODS

### Design, production, and purification of SOSIP trimers.

The B41 and BG505 trimers were based on the SOSIP.664 or SOSIP.v4 designs as specified in Results (46, 47). His-tagged variants contained 8 histidine residues attached via a GS-based linker to the C terminus of each gp41 component. The KG4 tag (see Results) contains the sequence KGKGKGK at the C terminus of each gp41 component. The various TCHE-tagged constructs are described in Table 2. The CZA97 SOSIP.v4.2-M6.IT trimer has been described elsewhere (82). The 16055 SOSIP.v8 trimer was based on an *env* gene described elsewhere (23), sequence modified for greater trimer stability by Philip Brouwer (Amsterdam University Medical Center, Amsterdam).

The nontagged BG505 SOSIP.v4.1 (clade A) and B41 SOSIP.v4.1 (clade B) trimers were expressed in stable CHO cell lines (49, 83). The lines were cultured in ProCHO5 medium (Lonza) supplemented with 1× GlutaMAX (Thermo Fisher Scientific) and 500 μg per ml of hygromycin B (Thermo Fisher Scientific).

The various tagged or otherwise sequence-modified variants of the B41 and BG505 SOSIP trimers, as well as the CZA97 and 16055 SOSIP trimers (see above), were expressed by cotransfecting FreeStyle 293-F cells (Thermo Fisher Scientific) with separate plasmids that express the *env* and *furin* genes (47, 84). In some cases, ExpiCHO-S cells (Thermo Fisher Scientific) were used. The FreeStyle 293-F cells (Thermo Fisher Scientific) were cultured in FreeStyle Expression medium (Thermo Fisher Scientific) containing 0.5× penicillin-streptomycin (Corning). The ExpiCHO-S cells (Thermo Fisher Scientific) were cultured with FectoCHO medium (PolyPlus transfection) supplemented with 1× GlutaMAX and 0.5× penicillin-streptomycin.

Trimers were purified from culture supernatants either by 2G12 affinity columns followed by size exclusion chromatography (SEC) or by PGT145 affinity columns (followed by SEC, if needed), using established methods (46). The B41 SOSIP.v4.1-His, B41 SOSIP.v4.1-A291T-His, and B41 SOSIP.v4.1-PADRE-v3 trimers were expressed in FreeStyle 293-F cells and purified by PGT145 affinity chromatography. The various KG4-tagged trimers were each expressed in ExpiCHO-S cells (Thermo Fisher Scientific) and purified by PGT145 affinity chromatography (85). All purified proteins were verified to be trimeric via blue native polyacrylamide gel electrophoresis (BN-PAGE) and NS-EM, as described previously (47). Protein concentrations were determined using a bicinchoninic acid (BCA)-based assay (Thermo Fisher Scientific).

### Coupling SOSIP trimers to IO-NPs.

SOSIP trimers were coupled to carboxylic acid-functionalized IO-NPs (Imagion Biosystems) with a 24-nm particle core diameter. For every 1.5 mg of particles, 500 to 800 μg of SOSIP trimers, at a concentration of at least 2 mg/ml, was used in the coupling reaction. In each production run, 1.5 mg of particles was added to a flat-bottom 1.5-ml Eppendorf tube (Watson Inc.), and the particles were activated by adding 17 μl of a stock solution containing 2 mg/ml 1-ethyl-3-(3 dimethylaminopropyl)carbodimide (EDC; Ocean Nanotech) and 1 mg/ml of *N*-hydroxysulfosuccinimide (NHS; Ocean Nanotech) in 2-(*N*-morpholino)ethanesulfonic acid (MES) buffer at pH 6.0, followed by continuous mixing for 30 min at room temperature. Sodium borate buffer, pH 8.0, was freshly prepared by mixing 250 ml of boric acid (12.4 g/liter; molecular weight [MW], 61.8) and 72.5 ml of sodium tetraborate (19.1 g/liter; MW, 381.4). A 2:1 volume of this borate buffer was then added to the activated particles, followed immediately by the addition of a SOSIP trimer stock that had been buffer exchanged into the same buffer. The mixture was incubated on a nutator for 2 h at room temperature, before addition of 10 μl of 200 mM glycine, pH 8.0, to quench the reaction. After a further 30-min incubation on the nutator, the IO-NPs were magnetically separated overnight by placing the reaction tubes into a magnetic separator (Ocean Nanotech) at 4°C. The supernatant was then removed, and the particles were resuspended in 1 ml of 75 mM NaCl, 20 mM Tris buffer, pH 8.0, before two further rounds of magnetic separation and resuspension in the same Tris-saline buffer, to fully separate particle-bound and soluble trimers. The final IO-NP trimer preparation was stored at 4°C.

The particles (25-μl aliquots) were first analyzed by SDS-PAGE to confirm trimer coupling. IO-NPs were magnetically separated from the supernatant for 1 h. After removal of the supernatant, the dry particles were resuspended in 8 μl of double-concentration NuPAGE LDS sample buffer (Invitrogen) supplemented with 500 mM dithiothreitol (DTT). The samples were then loaded onto a NuPAGE 4 to 12% bis-Tris gel for electrophoresis at 200 V for 30 min. The gel was washed 3 times with water for 10 min each time, exposed for 30 min to SimplyBlue SafeStain (Invitrogen), and destained overnight in 100 mM NaCl.

The protein concentrations in the IO-NP trimer suspensions were determined using a Macro BCA kit (Thermo Fisher Scientific). Protein standards of known concentrations from the kit (15 μl) were added in duplicate to 96-well microplates. Noncoupled IO-NPs (15 μl) were then added in an amount that mimicked what was present in the IO-NP trimer preparation. The IO-NP trimer preparation itself (15 μl) was mixed in duplicate wells with 15 μl of the above-described Tris-saline buffer. The BCA reagent (250 μl) was added for 1.5 h at 37°C, before the microplate was placed on a magnetic plate (Oz Biosciences) for 1 min to pellet the IO-NPs. A 225-μl aliquot of each supernatant (either protein standards or the test sample) was then transferred to a second microplate for measurement of the optical density at 562 nm (OD_562_).

The numbers of trimers per particle were estimated based on the number of IO-NPs present in the suspension, as stated by the manufacturer, and the protein content, as determined above. For example, using the peptidic molar mass of the trimer (2.14 × 10^5^) and a measured protein amount of 400 μg (in 1 ml), we made the following estimation: 4 × 10^−4^/2.14 × 10^5^ [g/(g/mol)] = 1.87 × 10^−9^ (mol), which yields 1.87 × 10^−9^ × 6.022 × 10^23^ [mol × (1/mol)] = 1.13 × 10^15^ trimers. When this value is divided by 5.56 × 10^13^, the total number of particles also present in 1 ml, we obtain an estimate that each IO-NP is carrying 20 trimers.

### Cryo-electron microscopy imaging of IO-NPs.

Samples of B41 SOSIP.v4.1-conjugated and unconjugated 25 nm IO-NPs were sonicated for 5 min in a water bath, before lauryl maltose neopentyl glycol (LMNG) was added at a final concentration of 10 μM. A 3-μl drop was then applied to a C-flat 2/2-50 copper, carbon-coated holey grid (Electron Microscopy Sciences), blotted, and plunge frozen in liquid ethane using a Vitrobot Mark IV robot (Thermo Fisher Scientific). The grids were imaged using a Thermo Fisher Scientific Talos F200C electron microscope operating at 200 keV and equipped with a Ceta 16M complementary metal oxide semiconductor camera (Thermo Fisher Scientific). Micrographs were collected using a nominal magnification of ×73,000, resulting in a pixel size of 1.98 Å at the specimen plane. IO-NPs were imaged over a range of defocus values of 0 to −4 μm. We found that the iron in the particles strongly interacts with the electron beam both by scattering and by diffraction, which complicated the detection of protein coupled to the IO-NP surface. In our experience, values close to the true focus provided the best visualization of protein species near the surface of the IO-NP core. Data collection was performed using Leginon software (86).

### Antibodies.

Monoclonal antibodies (MAbs) were obtained as gifts, produced in-house, or purchased from the following sources: VRC01, John Mascola; F105 and 17b, NIH AIDS Reagent Program; 3BNC117 and 8ANC195, Michel C. Nussenzweig; PG9, PGT121, PGT135, PGT145, and PGT151, International AIDS Vaccine Initiative; 2G12, Polymun Scientific; 19b, James Robinson; and BG1, BG18, and SF12, Pamela Bjorkman. The epitopes recognized by the human bNAbs 2G12, VRC01, PG9, PGT121, PGT135, and PGT145 and non-NAbs 17b, F105, and 19b used in this study are summarized elsewhere (https://www.hiv.lanl.gov/content/immunology/tables/ab_best_neutralizing_summary.html) (87, 88). MAbs RM19B1 and RM20A2 were isolated from BG505 SOSIP trimer-immunized macaques, will be more fully described elsewhere by Marit van Gils, Christopher Cottrell, Laura McCoy, and colleagues, and were produced in-house from plasmids provided by Marit van Gils. Briefly, plasmids expressing IgH and IgL were cotransfected into 293-F cells, and IgG was purified using protein A affinity columns. Rabbit MAb 10A was isolated from a BG505 SOSIP.664 trimer-immunized rabbit (89). The RM19B1 and RM20A2 MAbs recognize neoepitopes on the trimer base and are nonneutralizing, while 10A is a neutralizing MAb against a glycan hole formed by the absence of the N241 and/or N289 glycans. Rabbit MAbs 16D, C1, and C2 were similarly isolated by Marit van Gils, Christopher Cottrell, and Laura McCoy from B41 SOSIP trimer-immunized animals and produced in-house from plasmids, as noted above. MAbs 16D, C1 and C2 are described more fully elsewhere, where C1 is designated 45A and C2 is 48A (105). C1 and C2 are anti-base non-NAbs, while 16D is an NAb against the N289 glycan hole. The various MAbs were biotin labeled using an EZ-Link Sulfo-NHS-Biotin kit (Thermo Fisher Scientific) according to the manufacturer’s protocol. The rabbit immunization study was approved by the Institutional Animal Care and Use Committee (IACUC) at Covance Research Products (CRP), Inc. (Denver, PA). The macaque immunization was approved by the IACUC at the Wisconsin National Primate Research Center (Madison, WI).

### Antigenicity of soluble and IO-NP trimers by ELISA.

Nunc MaxiSorp 96-well flat-bottom plates (Thermo Fisher Scientific) were coated overnight at 4°C with 5.0 μg/ml of capture MAb (2G12, 16D, or 10A, as specified in Results) in 100 mM NaHCO_3_ (pH 8.0). The plates were washed three times with phosphate-buffered saline (PBS) plus 0.01% Tween 20 (PBST), before the wells were blocked in PBS with 3% bovine serum albumin (BSA) for 1 h at room temperature. Soluble SOSIP trimers (100-ng Env content) or IO-NP trimers (300-ng Env content) in PBS were then added for 2 h at 37°C. After washing as described above, biotin-labeled test MAbs, serially diluted in PBST, were added for 1 h at room temperature before removal by washing. Bound MAbs were detected using poly-horseradish peroxidase (HRP)–streptavidin (Thermo Fisher Scientific) at a 1:2,500 dilution in PBST for 1 h at room temperature. The color reaction was developed using the ABTS [2,2′-azinobis(3-ethylbenzthiazolinesulfonic acid)] peroxidase substrate (SeraCare) and terminated by adding SDS to a final concentration of 1%. The optical density was read at 405 nm. Curves were plotted using a sigmoid function with variable slope, after background subtraction, using GraphPad Prism (v8.0) software.

### Antigenicity of IO-NP trimers by NAb depletion assay.

Rabbit sera were heat inactivated at 56°C for 30 min and then diluted 1:10 in PBS (1:2 at Duke University Medical Center [DUMC]) and transferred in 200-μl aliquots (in 60-μl aliquots at DUMC) to flat-bottom 1.5-ml tubes (Watson Inc.). The SOSIP trimer-bearing and uncoupled IO-NPs were washed in PBS–10% fetal bovine serum (FBS) and resuspended in the same buffer before use. SOSIP trimer IO-NPs were added to a concentration of 100 μg/ml (Env content, as measured by a BCA-based assay), with the equivalent amount of uncoupled IO-NPs serving as a control, for 1 h of incubation at 37°C on a slow rocker (the final serum dilution at DUMC was 1:10). The tubes were then moved to a magnetic separating rack and incubated overnight at 4°C to pellet the trimer IO-NPs and their attached NAbs. The supernatant was then carefully removed while the tubes were still inserted in the magnetic separator and sterile filtered by centrifugation in Spin-X columns (Costar), before use in a neutralization assay (see below). Monoclonal antibodies were diluted to 125 μg/ml in PBS containing 10% FBS, before incubation with IO-NPs as described above.

### Modeling surface lysines on SOSIP trimers.

All ligands (including glycans) were removed from the 3.5-Å X-ray structure of B41 SOSIP.664 in complex with PGT124 Fab and 35O22 Fab (PDB accession number 6MCO), to leave a single gp120 plus gp41 chain. The MODELLER program (90) was used to generate a homology model that included an initially absent disordered segment plus the additional mutations inherent to the SOSIP.v4.1 design (50). The homology model was fit into EMD-8714 (a 4.7-Å cryo-EM ligand-free B41 SOSIP.664 structure), and symmetry copies were generated using the C3 symmetry of the map. The homology model was energy minimized by running Rosetta Relax (91) with EMD-8714 as a loose map constraint and with 3-fold symmetry imposed. The lowest-energy model was then used to add Man_9_ oligomannose glycans to each potential N-linked glycosylation site (PNGS) of B41 SOSIP.v4.1 using Rosetta SimpleGlycosylateMover and GlycanRelaxMover (92, 93). To speed up computation, each glycan was added individually. In total, 80 models were generated for each glycan. The best-scoring glycan models were sequentially added to the relaxed B41 SOSIP.v4.1 model, manually edited in Coot (94), and subjected to another round of Rosetta Relax. In total, 6 rounds of Rosetta Relax were performed to include all 30 glycans per protomer. Figures were generated using the UCSF Chimera program (95).

### B-cell activation by IO-NP trimers.

Ramos Burkitt’s lymphoma B cells stably expressing the BCR for the VRC01 bNAb were a gift from Daniel Lingwood (Massachusetts Institute of Technology, Boston, MA) (60). The line was cultured in RPMI 1640 medium (Thermo Fisher Scientific) containing 15% heat-inactivated fetal bovine serum (FBS; Gibco) and 1× penicillin-streptomycin and maintained at 37°C in 5% CO_2_. For experimental use, the cells were resuspended at 2 × 10^6^ per ml in RPMI 1640 medium supplemented with 15% fetal bovine serum (FBS) and the FLIPR Calcium 6 dye at the manufacturer-recommended final concentration (Molecular Devices) and then incubated in a 96-well U-bottom tissue culture plate (Costar) for 2 h at 37°C in air containing 5% CO_2_. The B41 SOSIP.v4.1 soluble or IO-NP trimers (10 μg of Env content) in 50 μl of the above-described medium, with the Calcium 6 dye still present, per the manufacturer’s recommendation, were added to a black 96-well plate with a transparent base (Thermo Fisher Scientific). The dye-loaded B cells (50 μl) were then added to the same wells, and the fluorescence signal (excitation at 494 nm, emission at 516 nm) was immediately recorded every 5 s for 3 min using an EnSpire multimode plate reader.

### Stability of IO-NP trimers in mouse serum.

Two 50-μg (Env content) aliquots of the B41 SOSIP.v4.1 IO-NP trimers were incubated overnight at 37°C in PBS that either contained or lacked 20% normal, non-heat-inactivated mouse serum. The particles were then washed twice with 500 mM NaCl, 20 mM Tris buffer and once with 75 mM NaCl, 20 mM Tris, pH 8.0, by magnetic pelleting and resuspension. The washed particles were finally resuspended in the latter buffer for subsequent analyses.

### Immunization and sampling of mice.

Male and female wild-type C57BL/6J mice of 6 to 8 weeks of age (The Jackson Laboratory) were equally distributed in the indicated experimental groups. The mice were housed in ventilated cages in environmentally controlled rooms. The mouse experiments were conducted at The Rockefeller University after approval by its IACUC. The mice were equally distributed in the experimental groups indicated above and were equally matched for gender. They were first immunized intraperitoneally (i.p.) with 50 μg of PADRE peptide (AKFVAAWTLKAAA; from Genaxxon Bioscience) in complete Freund’s adjuvant (Sigma), formulated according to the manufacturer’s instructions. After 25 days, the mice received, again by the i.p. route, the various B41 SOSIP.v4.1 trimer or trimer IO-NP immunogens (10 μg of Env protein in each case), which were formulated in the Sigma adjuvant system (Sigma) via the manufacturer’s instructions. Following this day 0 immunization, additional boosts with the same immunogens were given on days 17, 42, 75, and 136. The animals were bled 10 to 14 days after each immunization. All mice were housed in groups of five mice per cage and maintained on regular chow. One mouse in group 4 died before the fourth immunization, and two in group 3 died before the fifth (Fig. 7A). Group-specific serum pools included sera from those mice for the predeath time points.

### Mouse serum anti-trimer antibody ELISAs.

Anti-trimer antibody responses were determined by capture ELISA using the B41 SOSIP.v4.1-His trimer, essentially as reported elsewhere (52). The trimer was captured onto 96-well Ni-NTA plates (Qiagen Inc.) in Tris-buffered saline (TBS) plus 2% milk and 10% FBS. Mouse sera (non-heat inactivated) were titrated, and the trimer-bound antibodies were detected by an anti-mouse immunoglobulin secondary HRP-conjugated antibody (Bio-Rad). The serum dilution corresponding to an OD_450_ of 0.075 (the lowest value at least 2-fold higher than the background) was estimated from the titration curves and defined as the endpoint titer. Area-under-the-curve (AUC) analyses generally yielded outcomes similar to those of the titer determinations. A variant trimer in which the N289 glycan hole epitope had been knocked in via an A291T substitution was compared to the wild-type His-tagged B41 SOSIP.v4.1 trimer in some ELISAs. In this assay, the rabbit C1 Fab was added to the ELISA plates prior to addition of the sera in an attempt to block this non-NAb epitope at the trimer base.

To measure endpoint titers under limiting-antigen (LAg) and high-antigen (HAg) conditions, B41 SOSIP.v4.1-His trimers were captured onto Ni-NTA plates at 0.2-μg/ml and 1.5-μg/ml concentrations, respectively. The LAg concentration was identified by titrating the trimer input, followed by detection with 2G12 (1 μg/ml), and identifying the lowest concentration (i.e., 0.2 μg/ml) that gave a quantifiable signal over the background (OD = 0.5). The total IgG samples from bleed 5 were then titrated, and the endpoint titers were estimated as described above.

To measure antibodies to the PADRE TCHE tag on trimer immunogens, the GGSGGSPMGLPAKFVAAWTLKAAAPMGLPGGSGGSKKGGHHHHHH peptide was synthesized by Thermo Fisher Scientific, and the tag sequence was followed by a hexa-His motif. After attachment to Ni-NTA plates via the His tag, the ELISA procedure was performed as described above.

In the anti-trimer base competition ELISA, B41 SOSIP.v4.1 trimers were captured onto the wells via Galanthus nivalis lectin (Sigma) (96). The anti-289 glycan hole NAb epitope assay used a His-tagged version of the same trimer, which was captured onto Ni-NTA plates via the tag. In both cases, mouse sera or group-specific serum pools (non-heat inactivated) were added at a 1:100 dilution (or titrated) for 1 h. The pools were prepared by mixing equal volumes of each individual serum sample. As a positive control for competition, the unlabeled version of the biotin-labeled detection MAb was used at 2 μg/ml. The biotin-labeled MAb (final concentration 0.2 μg/ml for C1 or C2 to trimer base and 0.5 μg/ml for 16D to 289 glycan hole) was then added for a further 30 min. Bound MAbs were then detected using streptavidin-HRP (Pierce) at a 1:2,500 dilution. Background control values, determined by assays where all steps were identical to those described above, except that no trimer was present, were subtracted from all experimental values (i.e., values from assays in which trimer was present). The binding of biotin-labeled MAb in the absence of mouse serum (buffer control) was defined as 100%. The residual binding was then calculated as follows: percent residual binding = (OD_450_ competitor/OD_450_ buffer)·100, where OD_450_ competitor is the signal derived from wells where mouse serum or the unlabeled MAb was added as the competing agent, and OD_450_ buffer is the signal from wells with buffer but no competing agent. Finally, the percent competition was calculated as 100 (percent) − residual binding (percent). In these assays, 50% inhibition of biotin-labeled MAb binding occurred at the following concentrations of the same unlabeled MAb: C1, 0.043 μg/ml; C2, 0.11 μg/ml; 14e, 1.05 μg/ml; 19b, 0.37 μg/ml; and 16D, 0.26 μg/ml.

### NS-EM imaging of trimer-Fab complexes.

Fab fragments were prepared from IgG samples derived from pools of the terminal bleed (day 189) mouse sera for groups 1 and 2 using a Pierce Fab preparation kit (Thermo Fisher Scientific) following the manufacturer’s protocol. The purified Fab fragments were buffer exchanged and concentrated into TBS using Amicon Ultra 0.5-ml centrifugal filters with a 10-kDa cutoff (Millipore Sigma). The resulting Fab preparations (200 μg) were then spiked with 10 μg of B41 SOSIP.v4.1 trimer and incubated overnight at room temperature to form trimer-Fab complexes. Unbound Fab was then removed by SEC using a Superose 6 Increase 10/300 column (GE Healthcare). The flowthrough fractions containing the complexes were then pooled and concentrated using a 100-kDa-cutoff centrifugal filter (EMD Millipore). The final complex concentration was adjusted to ∼40 μg/ml for NS-EM imaging. The complexes were applied to glow-discharged, carbon-coated 400-mesh copper grids, followed by the addition of 3 μl of 2% (wt/vol) uranyl formate stain for 45 to 60 s and blot drying. The staining procedure was then repeated using a second 3-μl aliquot, followed by blotting. The stained grids were stored under ambient conditions until used for imaging. Images were collected via Leginon software using a Tecnai T12 electron microscope operated at 120 kV and a ×52,000 magnification (97). In all cases, the electron dose was 25 e^−^/Å^2^. Particles were picked from the raw images using the DoG Picker program and placed into stacks using Appion software (98). The two-dimensional (2D) reference-free alignment procedure was performed using iterative multivariate statistical analysis/multireference alignment. The particle stacks were then converted from IMAGIC to RELION-formatted Medical Research Council (MRC) stacks and subjected to RELION 2.1 2D and three-dimensional (3D) classification (99).

### Env-pseudotyped viruses.

The Env-pseudotyped viruses used for neutralization assays at Weill Cornell Medical College (WCMC) (BG505.T332N, B41, and MW965.26) were prepared in 293T cells essentially as described previously (46). The B41 N289 glycan mutant virus contained an A291T substitution to knock in a glycan and block the autologous NAb epitope at that site (52). Both the wild-type and the mutant B41 viruses also contained an R315Q sequence change in V3 to match the B41 SOSIP trimer immunogen (46).

### HIV-1 neutralization by mouse sera and purified IgG.

The Tzm-bl cell-based assay using Env-pseudotyped viruses performed at WCMC has been described previously (46, 47, 59). Tzm-bl cells were cultured in high-glucose Dulbecco’s modified Eagle medium (DMEM; Corning) supplemented with 1× penicillin-streptomycin and 10% heat-inactivated FBS and maintained at 37°C in 5% CO_2_. Briefly, serially diluted sera (non-heat inactivated) were incubated with an Env pseudovirus for 1 h at 37°C, before adding the mixture to the cells. Neutralization was defined as the percent reduction of the infectivity obtained in the absence of serum.

IgG was purified from mouse sera using protein A plus protein G binding, as described previously (100). Briefly, serum was diluted 25-fold in PBS and passed through a Sepharose column containing a 1:1 mixture of protein A (catalog number 17-5138-01; GE Healthcare) and protein G (catalog number P3296; Sigma-Aldrich). The bound IgG was eluted with 100 mM glycine, pH 3, and immediately neutralized using 2 M Tris buffer, pH 8. The eluate was diluted 2.5-fold with PBS and centrifuged three times using Vivaspin 6 (10-kDa-cutoff; GE Healthcare) columns. The recovered IgG was reconstituted to the original volume in PBS. IgG recovery was measured by ELISA (Molecular Innovations Inc.). The purified IgG samples were then tested for NAbs at the Duke University Medical Center (DUMC), again using the Tzm-bl cell assay and Env-pseudotyped viruses (101).

### MBL interactions with IO-NP trimers.

The VRC01 bNAb (500 ng) was coated overnight onto ELISA wells in 100 mM NaHCO_3_ buffer. The wells were then blocked with 3% BSA in PBS. Soluble or IO-NP-coupled B41, BG505, or 16055 SOSIP.v4.1-KG4 trimers or the nontagged B41 trimer (each at 150 ng in 100 μl) was added for 2 h at 37°C. Various concentrations of recombinant truncated mouse MBL (MyBiosource Inc.), full-length mouse MBL (Sino-Biological), truncated or full-length human MBL (MyBiosource Inc.), or normal, non-heat-inactivated mouse or human serum (Sigma-Aldrich) were then added for 1 h. In some experiments, the biotin-labeled 2G12 bNAb was titrated as a control to assess trimer loading onto the ELISA plate wells. After washing the plates, bound mouse or human MBL was detected using a biotinylated goat anti-human or anti-mouse MBL polyclonal antibody (0.5 μg per ml; R&D Systems). Bound 2G12 was detected using streptavidin-HRP (1/2,500). Similar MBL reactivity was seen when the B41 IO-NP trimers were captured via PGT145 or VRC01 or coated directly onto the wells. An alternative ELISA for mouse or human serum MBL reactivity with His-tagged soluble B41 SOSIP.v4.1 trimers was performed using the trimer-capture conditions described separately for His-tagged trimers and Ni-NTA plates, followed by MBL detection, as outlined above.

In the MAb competition assay, recombinant full-length mouse MBL (0.5 μg in 50 μl) was added to VRC01-immobilized B41 SOSIP.v4.1-KG4 IO-NP trimers for 1 h. Biotin-labeled MAbs at twice the final concentration were then added in a 50-μl volume for a further 30 min, and the wells were then washed three times with PBST. Alternatively, normal mouse serum (10% dilution in PBS) or purified mouse IgG (2 mg/ml in PBS; Sigma-Aldrich) was added instead of MBL. Bound MAbs were detected using streptavidin-HRP (1/2,500). In control experiments, normal mouse serum (2% dilution in 50 μl of PBS) was incubated for 30 min with an equal volume of 100 mM d-mannose or d-galactose (Sigma-Aldrich). The mixture (1% serum plus 50 mM d-mannose or d-galactose) was then added to VRC01-captured B41 SOSIP.v4.1-KG4 IO-NP or soluble trimers for 1 h. Bound MBL was then detected as described above.

### Statistical analyses.

Comparisons of two groups were performed by the two-tailed Mann-Whitney test, and comparisons of more than two groups were performed by the Kruskal-Wallis test with Dunn’s multiple-comparisons posttest. Correlations were analyzed by calculating Spearman *r* coefficients for strength and two-tailed *P* values for significance. In ELISAs, means were calculated from two duplicate titrations, and the standard error of the mean (SEM) values are displayed as error bars.

## Figures and Tables

**FIG A8:**
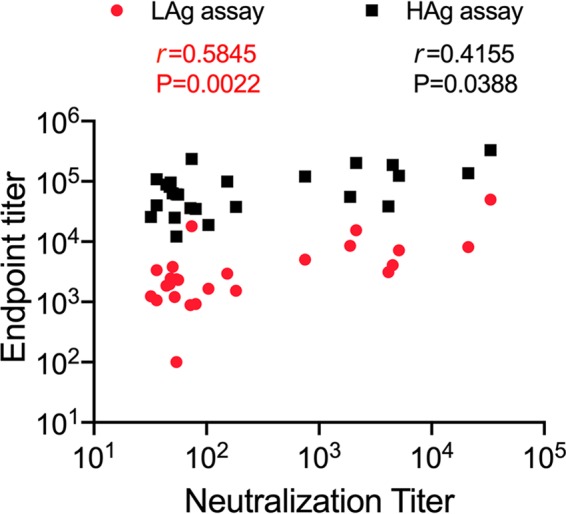
Relative intrinsic affinities of mouse serum anti-trimer antibodies. Correlation between ELISA binding and neutralization titers. Mouse IgG from bleed 5 was titrated against the B41 SOSIP.v4.1-His trimer captured at 1.5 μg/ml (HAg assay; black squares) or 0.2 μg/ml (LAg assay; red circles). The resulting endpoint titers are plotted on the *y* axis against the B41 neutralization titers (Fig. 7A) on the *x* axis. The *r* and *P* values of the Spearman correlations for the two comparisons are recorded.

## APPENDIX

An antigenicity analysis of B41 SOSIP trimers before and after IO-NP attachment is shown in Fig. 1. A similar analysis implied that the BG505 SOSIP.664 trimers were not coupling in an appropriate orientation (Fig. A1). Although the BG505 SOSIP.664 IO-NP trimers presented the PGT145 and VRC01 epitopes, the V3 non-NAb epitope for 19b was more highly exposed than it was on the soluble trimer, and trimer base epitopes for rhesus macaque non-NAbs RM19B1 and RM20A2 were fully accessible (Fig. A1A). However, the antigenicity profile of the more stable BG505 SOSIP.v4.1 IO-NP trimers resembled that of the same soluble trimers for bNAbs (PGT145, PGT151, VRC01), non-NAbs (F105, 17b, 19b), and the autologous N241 glycan hole NAb 10A. In contrast, the RM19B1 and RM20A2 trimer base epitopes were exposed on soluble SOSIP.v4.1 trimers but occluded on the IO-NPs (Fig. A1B). In summary, BG505 SOSIP.664 trimers couple to IO-NPs efficiently but in an ill-defined orientation, while their SOSIP.v4.1 counterparts attach less well but predominantly via their base.

The B41 SOSIP.v4.1 trimer has 102 lysine residues. When considering the protein alone, many of these residues have a solvent-accessible side chain amine that could, in principle, serve as sites of attachment to IO-NPs (Fig. 1A and A2A). The number of lysine residues available for NHS-ester linker chemistry is substantially reduced when the 90 potential N-linked glycosylation sites (PNGS) are considered. A hybrid model of the fully glycosylated trimer suggests that all lysine residues at the apex are completely glycan obscured (Fig. A2A). While our model does not take into account glycan motions, it further suggests that most lysines along the top and sides of the trimer are unlikely to participate in cross-linking due to the shielding effects of nearby glycans. For example, the K231 residue is 83.7% conserved across HIV-1 strains. Its ε-amino group is exposed in an opening surrounded by a glycan cluster (N234, N241, N295, N339, N448), with N241 being particularly close (Fig. A2B). Trimers are coupled to IO-NPs via a zero-length linker, i.e., a short amide bond to the particle surface (Fig. 1A). N-linked glycans have several degrees of freedom in terms of torsion angles, but we estimate that the average distance from the peptide asparagine residue to the tip of a Man_9_ glycan is ∼20 Å. Hence, it is quite unlikely that K231 could be a site for trimer attachment to IO-NPs (Fig. A2B).

The trimer model further suggests that the glycan-free base of each protomer exposes three closely proximal lysines, K33, K34, and K502, for coupling to IO-NPs (Fig. A2A). While N-terminal sites K33 and K34 are quite variable (<20% conservation), the C-terminal K502 is highly conserved (82.8%). Thus, K502 may be a primary attachment point for multiple trimer genotypes. The B41 SOSIP-E64K.M1M7 trimer has glycans knocked in at V3 positions 306 and 314 to further shield the V3 region, including nearby lysine residues (102). The efficient coupling of this trimer to IO-NPs is additional evidence that lysine residues in or near V3 are not attachment sites (Table 1).

We used a similar model to study the lysine residues exposed on the surface of BG505 SOSIP.664 trimers (Fig. A2C and D). Unlike B41, only lysine K502 is accessible on the BG505 trimer base and the N618 glycan is closer than it is on its B41 counterpart, N616 (Fig. A2C and D). The differences may account for the lower coupling stoichiometry for BG505 versus B41 SOSIP.v4.1 trimers (Table 1). A lysine-rich patch on the BG505 trimer flank involving the C2 and C3 regions flanking V3 may be exposed by the absence of the N241 glycan (Fig. A2E). This patch could explain the atypical attachment of BG505 SOSIP.664 trimers that is implied by antigenicity assays (Fig. A1A). The model cannot distinguish between SOSIP.664 and SOSIP.v4.1 trimers, but the V3 lockdown effect of the v4.1 mutations may reduce the accessibility of these C2 and/or C3 lysines and, hence, allow BG505 SOSIP.v4.1 trimers to attach via their base (Fig. A1B). As was found with B41 SOSIP-E64K.M1M7, the BG505 SOSIP-E64K-M1M7 IO-NP trimers attached efficiently to IO-NPs (Table 1). These trimers have glycans knocked in to shield the V3 region, including nearby lysine residues (102). The implication is, again, that lysine residues in or near V3 are not primary sites of trimer attachment to IO-NPs.

The lysine-rich KG4 tag to attach SOSIP trimers to IO-NPs via their base is described in Fig. 3 and Table 1. Antigenicity analyses by ELISA showed that BG505 SOSIP.v4.1-KG4 trimers are coupled to IO-NPs in an appropriate orientation (Fig. A3B), which is also the case for KG4-tagged trimers from the B41, 16055, and CZA97 genotypes (Table 1; Fig. A3C to E).

The ability of B41 SOSIP.v4.1-KG4 IO-NP trimers to bind and remove NAbs in a solution phase assay is demonstrated in Fig. 4. Similarly, incubation with BG505 SOSIP.v4.1-KG4 IO-NP trimers ablated the BG505.T332N virus-neutralizing capacity of the VRC01 bNAb, rabbit MAb 10A, and two serum samples from BG505 SOSIP.664 trimer-immunized rabbits (Fig. A4A and B) (52). The same IO-NP trimers also depleted the MW965.26 virus-neutralizing ability of VRC01 and rabbit serum NAbs (Fig. A4C).

The results of a mouse immunogenicity study using soluble and IO-NP trimers based on the B41 SOSIP.v4.1-PADRE-v3 design are described in Fig. 6. We did not use a KG4-tagged version, in part because of difficulties involved in incorporating the PADRE-v3 and KG4 tags into the same construct. On a BN-PAGE gel, the soluble SOSIP.v4.1-PADRE-v3 trimer migrated as an appropriately sized band with no other Env forms visible (Fig. A5A). The ELISA antigenicity profiles of the same soluble trimers were comparable to those of the wild type, except for a substantial reduction in the binding of anti-base MAbs C1 and C2, which presumably reflects an epitope-occluding effect of the PADRE-v3 tag (Fig. A5B; cf. Fig. 1D). The B41 SOSIP.v4.1-PADRE-v3 and wild-type trimers coupled to IO-NPs at similar stoichiometries and with comparable ELISA antigenicity profiles (Table 1; Fig. A5B; cf. Fig. 1B to D).

We routinely found that trimer IO-NPs are stable for multiday periods in neutral pH buffers at 4°C, as judged by antigenicity assays and gel profiles. To further test their stability before conducting a mouse immunogenicity study, we incubated B41 SOSIP.v4.1 IO-NP trimers overnight at 37°C in the presence of 20% non-heat-inactivated mouse serum. The antigenicity of the PGT145 and VRC01 bNAb and F105 non-NAb epitopes was unchanged, implying that the immunogen was stable under these conditions (Fig. A6).

The primary antibody response outcomes of a mouse immunogenicity experiment are described in Fig. 6 and 7 and Table 3. Additional serology analyses discussed in the Results section are presented in Fig. A7.

To determine the relative intrinsic affinities of the mouse serum antibodies and compare them with the NAb assay data, we performed limiting-antigen (LAg) and high-antigen (HAg) anti-trimer ELISAs on bleed 5 IgG samples (103, 104). In both ELISAs, the endpoint anti-trimer antibody titers were higher for soluble trimer groups than for IO-NP trimers, but the difference was more marked in the LAg assay than in the HAg assay (*P* = 0.019 versus *P* < 0.0001) (Fig. A8). The nonparametric Spearman correlation between B41 NAb titers and the LAg assay endpoint titers for bleed 5 samples was also stronger for the LAg assay than for the HAg assay (Fig. A8). These differences are compatible with affinity maturation processes being more advanced in the soluble trimer than in the IO-NP trimer groups. Of note is that intrinsic affinities should influence HIV-1 neutralization more strongly than avidity (62).
